# Comprehensive Analysis of Nasal Polyps Reveals a More Pronounced Type 2 Transcriptomic Profile of Epithelial Cells and Mast Cells in Aspirin-Exacerbated Respiratory Disease

**DOI:** 10.3389/fimmu.2022.850494

**Published:** 2022-03-28

**Authors:** Christine Bangert, Sergio Villazala-Merino, Martin Fahrenberger, Thomas Krausgruber, Wolfgang M. Bauer, Victoria Stanek, Nicholas James Campion, Tina Bartosik, Tamara Quint, Guenther Regelsberger, Verena Niederberger-Leppin, Christoph Bock, Sven Schneider, Julia Eckl-Dorna

**Affiliations:** ^1^ Department of Dermatology, Medical University of Vienna, Vienna, Austria; ^2^ Department of Otorhinolaryngology, Medical University of Vienna, Vienna, Austria; ^3^ Center for Integrative Bioinformatics Vienna (CIBIV), Max Perutz Labs, University of Vienna and Medical University of Vienna, Vienna, Austria; ^4^ CeMM Research Center for Molecular Medicine of the Austrian Academy of Sciences, Vienna, Austria; ^5^ Division of Neuropathology and Neurochemistry, Department of Neurology, Medical University of Vienna, Vienna, Austria; ^6^ Institute of Artificial Intelligence and Decision Support, Center for Medical Statistics, Informatics, and Intelligence Systems, Medical University of Vienna, Vienna, Austria

**Keywords:** aspirin-exacerbated respiratory disease, chronic rhinosinusitis with nasal polyps, type 2 immunity, mast cells, scRNA-seq analysis

## Abstract

Chronic rhinosinusitis with nasal polyps is affecting up to 3% of Western populations. About 10% of patients with nasal polyps also suffer from asthma and intolerance to aspirin, a syndrome called aspirin-exacerbated respiratory disease. Although eosinophilic inflammation is predominant in polyps of both diseases, phenotypic differences in the tissue-derived microenvironment, elucidating disease-specific characteristics, have not yet been identified. We sought to obtain detailed information about phenotypic and transcriptional differences in epithelial and immune cells in polyps of aspirin-tolerant and intolerant patients. Cytokine profiles in nasal secretions and serum of patients suffering from aspirin-exacerbated respiratory disease (n = 10) or chronic rhinosinusitis with nasal polyps (n = 9) were assessed using a multiplex mesoscale discovery assay. After enrichment for immune cell subsets by flow cytometry, we performed transcriptomic profiling by employing single-cell RNA sequencing. Aspirin-intolerant patients displayed significantly elevated IL-5 and CCL17 levels in nasal secretions corresponding to a more pronounced eosinophilic type 2 inflammation. Transcriptomic profiling revealed that epithelial and mast cells not only complement one another in terms of gene expression associated with the 15-lipoxygenase pathway but also show a clear type 2-associated inflammatory phenotype as identified by the upregulation of *POSTN*, *CCL26*, and *IL13* in patients with aspirin-exacerbated respiratory disease. Interestingly, we also observed cellular stress responses indicated by an increase of *MTRNR2L12*, *MTRNR2L8*, and *NEAT1* across all immune cell subsets in this disease entity. In conclusion, our findings support the hypothesis that epithelial and mast cells act in concert as potential drivers of the pathogenesis of the aspirin-exacerbated respiratory disease.

## Introduction

Chronic rhinosinusitis is a heterogeneous disease frequently associated with the burdensome occurrence of nasal polyps (CRSwNP). Being characterized by nasal obstruction and loss of smell, this entity affects up to 3% of the Western population (1, 2). While asthma can be observed in approximately 50% of patients with CRSwNP (3), its presence is mandatory in aspirin-exacerbated respiratory disease (AERD), a symptom triad additionally characterized by nasal polyps and aspirin intolerance (4). Well-explored AERD-underlying pathomechanisms include a decrease in the anti-inflammatory mediator prostaglandin E2 upon aspirin-induced cyclooxygenase-1 inhibition and, subsequently, a preponderance of the cyclooxygenase-2 dependent proinflammatory mediator prostaglandin D2 and its metabolites (5, 6). The metabolism of arachidonic acid further leads to an accumulation of leukotrienes and an extensive eosinophilic mucosal type 2 (T2) inflammation in the lower and upper airways (6). A varying degree of eosinophilic infiltration associated with a strong T2 immune response is a typical feature of nasal polyps in both CRSwNP without aspirin intolerance and AERD (7). It has been proposed that mechanisms driving the pathology of CRSwNP and AERD are similar but differ in their intensity leading to a more severe immune reaction in AERD patients (6). Recent transcriptomic data analyzing potential differences in the arachidonic acid metabolic pathway of nasal polyps in AERD as compared to CRSwNP patients revealed a significant elevation of 15-lipoxygenase (15-LO) predominantly in epithelial cells of AERD (8). Being centrally involved in the pathogenesis of chronic rhinosinusitis, epithelial cells are reduced in their diversity in response to a chronic T2 immune environment resulting in severe impairment of the physiological barrier function (9). Mast cells (MCs) as an important source of high concentrations of proinflammatory mediators such as histamine, cysteinyl leukotriene E4, or prostaglandin D2 further contribute to AERD-specific pathophysiology (5, 6). Of note, the number of infiltrating MCs in polyps of AERD patients is significantly larger as compared to polyps from aspirin-tolerant CRSwNP patients (10). Further analysis on a molecular single-cell level identified transcriptionally and phenotypically distinct clusters of MCs including intraepithelial tryptase+/chymase− MC_T_ as well as subepithelial tryptase+/chymase+ MC_CT_ in both AERD and CRSwNP patients (10). To date, several studies have analyzed specific polyp-derived cell subsets or AERD-related pathways (8, 10, 11), but a comprehensive transcriptional analysis of the entire microenvironment comparing AERD and aspirin-tolerant CRSwNP to further elucidate disease-specific pathomechanisms is still missing. To address this shortcoming, we subjected polyp tissue of patients with CRSwNP and AERD to a detailed transcriptomic analysis by single-cell RNA sequencing (scRNA-seq) supplemented by information on a broad panel of cytokines in nasal secretions and serum. By doing so, we obtained thorough information about the nasal cytokine signature and phenotypic diversity in epithelial cells and different immune cell subsets infiltrating polyps of aspirin-tolerant CRSwNP and aspirin-intolerant AERD patients.

## Material and Methods

### Study Design and Patients’ Characteristics

After approval of the ethics committee of the Medical University of Vienna (EK No. 1956/2018) and registration with clinicaltrials.gov (NCT03848156), the study was conducted at the Department of Otorhinolaryngology of the Medical University of Vienna. Written informed consent was obtained from each patient before study inclusion according to the Declaration of Helsinki. Of 28 patients screened, 23 adult patients of Caucasian origin with guideline-confirmed CRSwNP without aspirin intolerance (n = 10) or AERD (n = 13) were included (4, 12). In AERD patients, aspirin intolerance was confirmed by either provocation testing or documented medical history of previous intolerance reaction to aspirin (4). In one AERD patient, there were not enough cells available for cell sorting after the preparation of single-cell suspension. After cell quality control of the samples, two AERD patients (#6 and #26) and one CRSwNP patient (#19) were excluded (**Figures S1A, B**), and subsequently, 19 patients were selected for analysis. Patients’ characteristics are displayed in **Tables 1**, **S1**. All patients maintained their standard treatment regimen to ensure real-life conditions. Nasal secretions, nasal polyp biopsies, and serum samples were obtained from all patients and immediately stored on ice until further processing.

**Table 1:** Study population.

		CRSwNP (n = 9)	AERD (n = 10)
Sex	M/F	7/2	3/7
Age (years)	Median	53	54
	Range	25–75	25–69
Asthma Allergy	Y/N	2/7	10/0
	Y/N	4/5	6/4
Total IgE (kU/L)	Median	45.8	148.5
	Range	9.5–422	47.3–470
Total Snot-20 GAV score	Median	30	38
	Range	15–47	16–54
Total NPS	Median	4	5.5
	Range	2–8	2–8
Nasal topical corticosteroid	Y/N	5/4	10/0
Previous polyp surgery	Y/N	3/6	10/0

CRSwNP, Chronic rhinosinusitis with nasal polyps; AERD, aspirin-exacerbated respiratory disease; M, male; F, female; Y, yes; N, no; Snot-20 GAV, Sino-Nasal Outcome Test 20, German adapted version; NPS, Nasal Polyp Score.

### Nasal Sampling

Nasosorptions (Nasosorption FX-I, Hunt Developments (UK) Limited, Midhurst, UK) were applied for the collection of nasal secretions (**Figure S2**) and processed as previously described (13). Prior to polyp biopsy, patients received local anesthetics and decongestants. Biopsies were taken during endoscopy and kept in phosphate-buffered saline (PBS) on ice until further processing. One part of the nasal biopsy was embedded in Tissue-Tek^®^ O.C.T Compound (Sakura, Tokyo, Japan) and frozen at −80°C for later microscopic analysis (**Figure S2**). The other part was enzymatically digested for 1 h at 37°C using RPMI-1640 medium (Thermo Fisher Scientific, Waltham, MA, USA) supplemented with 2.8 mg/ml of Collagenase IV, Thermo Fisher Scientific) and 0.03 mg/ml of DNAse (Merck, Burlington, MA, USA). Thereafter, single-cell suspensions were obtained by passing tissues through a 70-µm cell strainer (BD Biosciences, Franklin Lakes, NJ, USA) and washing twice with RPMI-1640 medium supplemented with 20% fetal bovine serum (FBS, Thermo Fisher Scientific). Cells were then blocked with 10% mouse serum (Thermo Fisher Scientific) in MACS buffer (Miltenyi Biotec, Bergisch Gladbach, Germany) on ice for 5 min. Preparing cell suspensions for fluorescence-activated cell sorting (FACS), cells were stained with anti-human CD15 FITC (clone W6D3, BioLegend, San Diego, CA, USA), anti-human CD94 PE (clone DX22, BioLegend), anti-human CD14 APC (clone HCD14, BioLegend), anti-human HLA-DR BV421 (clone L243, BioLegend), anti-human CD45 PE-CF594 (clone HI30, BD Biosciences), anti-human CD11c PE-Cy7 (clone 3.9, BioLegend), anti-human CD20 APC-Cy7 (clone 2H7, BioLegend), and anti-human CD3 BV605 (clone SK7, BioLegend) with an incubation period of 30 min on ice under light protection. After staining, cells were washed twice with MACS buffer and resuspended in a final volume of 100 µl of MACS buffer containing 7-AAD (Merck).

Cells were then subjected to FACS (FACSAria III, BD Biosciences) (**Figure S2**) for the enrichment of CD45+ leukocyte subsets to obtain comparable numbers between all samples, allowing for comparison of small leukocyte cell populations as previously described (14).

### Mesoscale Discovery Assay

Mediator concentrations within nasal secretions and serum samples were measured using electrochemiluminescence technology through the use of the mesoscale discovery (MSD) U-Plex assay as described by the manufacturer (https://www.mesoscale.com/en). The following cytokines were analyzed: BAFF, Eotaxin, Eotaxin 3, G-CSF, GM-CSF, IFN-γ, IL-10, IL-12, IL-12p70, IL-13, IL-15, IL-16, IL-17A, IL-1α, IL-1RA, IL-1B, IL-2, IL-3, IL-4, IL-5, IL-6, IL-7, IL-8, IL-9, IL-21, IL-22, IL-25, IL-27, IL-33, TARC, TNF-α, TNF-β, TSLP, and VEGF-A. Briefly, custom cytokine plate kits were purchased from MSD (Rockville, MA, USA). First, linker-antibody solutions were prepared according to the protocol and then used to coat the U-Plex plate (50 μl per well), and the plates were left shaking overnight at 2°C–8°C.

To prepare calibrator standards, the calibrator was diluted 1/10 in the provided metabolic assay working solution, and 8 standards were subsequently created with ¼ dilution steps. After standard preparation was complete, the coated plates were washed 3 times with 150 μl of PBS-T (PBS 0.05% Tween 20) in each well. Standard or sample measuring 50 μl was added to each well. The plate was subsequently sealed and incubated while shaking for 2 h at room temperature. Thereafter, plates were washed 3 times with PBS-T, and 50 μl of the provided detection antibody solution was added to each well followed by another incubation period of 1 h while shaking. The plate was again washed 3 times with PBS-T, and 150 μl of the kit supplied MSD GOLD read buffer was added to each well. The plates were then analyzed using the MESO SECTOR S 600 plate reader (MSD), and cytokine concentrations were calculated using the DISCOVERY WORKBENCH software (MSD, Rockville, MA, USA). Data were analyzed using Graph Pad Prism software (version 9.3.0). Cytokine concentrations were log-transformed for analysis to ensure their fitting to a normal distribution. Statistically significant differences (p-value <0.05) were determined using multiple unpaired t-tests with Welch’s correction per row. If multiple cytokines were analyzed simultaneously, the Holm–Sidak method was applied for correction, and adjusted p-values are displayed.

### Immunofluorescence and Quantitative PCR

Immunofluorescence was performed on frozen O.C.T-embedded samples. Anti-human IL-5Rα (Benralizumab, AstraZeneca, Cambridge, UK) was labeled with AlexaFluor488 (Thermo Fisher Scientific) according to the manufacturer’s instructions and then together with anti-human eosinophilic major basic protein (MBP; clone BMK-13, Bio-Rad, Hercules, CA, USA) and anti-human c-KIT (CD117, polyclonal, Agilent, Santa Clara, CA, USA) applied to cryosections overnight. Thereafter, slides were washed, and secondary antibodies anti-mouse AlexaFluor546 (Thermo Fisher Scientific) and anti-rabbit AlexaFluor405 (Thermo Fisher Scientific) were applied for 1 h at room temperature. Slides were counterstained with DRAQ5™ (BioLegend) and mounted with prolonged antifade gold (Thermo Fisher Scientific). Images of slides were acquired using a confocal laser scanning microscope (Nikon, Tokyo, Japan; ×60 magnification). For quantification, images comprising >1 × 10^3^ cells were acquired near the mucosal surface and counted using ImageJ. Cells of the mucosa were discounted due to the high background, and only submucosal cells were included. Nuclei were counted using the “analyse particles” function after appropriate thresholding. Cells staining positive for one or multiple epitopes were counted using the “cell counter” function in a stack in ImageJ.

mRNA for quantitative PCR was isolated from snap-frozen tissue using TRIzol reagent, transcribed to cDNA, and analyzed using TaqMan^®^ real-time PCR assays (TSLP, IL33, MTRNR2L8, NEAT) as previously described (14). Data were analyzed using Graph Pad Prism software (version 9.3.0); statistically significant differences were determined using the Mann–Whitney test.

### Droplet-Based Single-Cell RNA Sequencing

Cells sorted from AERD and CRSwNP biopsies after mechanical and enzymatic isolation were used to generate libraries for scRNA-seq (Gene Expression Omnibus identifier: GSE196169). Such libraries were generated using the Chromium Controller and Single Cell 3′ Library & Gel Bead Kit v2 (samples 1–9) and v3 (samples 10–19) (10x Genomics, Pleasanton, CA, USA) according to the manufacturer’s protocol and as previously described (15). Briefly, up to 18,000 sort-purified cells from each sample were suspended in reverse transcription reagents, along with gel beads, and then segregated into aqueous nanoliter-scale gel bead-in-emulsions. After gel bead-in-emulsions reverse transcription, single-cell droplets were broken, and the single-strand cDNA was isolated and amplified. Subsequently, the amplified cDNA was fragmented, end-repaired, A-tailed, and index adaptor ligated followed by post-ligation amplification. The sequencing-ready libraries were sequenced by the Biomedical Sequencing Facility at the CeMM Research Center for Molecular Medicine of the Austrian Academy of Sciences, using the Illumina HiSeq 3000/4000 platform and the 75-bp paired-end configuration.

### Secondary Analysis of Single-Cell RNA Sequencing

#### Single-Cell Data Analysis

The raw sequencing reads were aligned to the reference genome GRCh38 (16) and demultiplexed using Cell Ranger 3.0.2 (17). The resulting count-matrices were then imported into R for further analysis using the Seurat package v3 (18).

#### Cell Quality Control

As is common practice, cells were filtered based on their count depths, their number of expressed genes, and the percentage of mitochondrial reads present. The cutoffs for these three metrics were manually chosen for each sample based on the distribution within the sample. Samples 6, 19, and 26 were removed from analysis due to their abnormal distributions in either count depths or percentage of mitochondrial reads (**Figures S1A, B**).

#### Data Processing and Integration

The remaining 19 samples were normalized with Seurat’s NormalizeData function using log normalization, and the 2000 genes with the highest variance of expression across samples were identified through FindVariableFeatures. Subsequently, the samples were combined into one dataset using the FindIntegrationAnchors and IntegrateData functions with the first 20 canonical correlation analysis (CCA) dimensions (18) and otherwise default parameters. The integrated dataset was then z-score scaled, and principal components were calculated. The UMAP transformation was then calculated based on the first 20 principal components.

#### Clustering

The cells from all samples were clustered jointly through the FindClusters function using smart local moving (19) on the first 20 principal components with a resolution parameter of 0.6. Marker genes for the resulting clusters were identified afterward using FindAllMarkers function, which uses the Wilcoxon rank-sum test internally, and cell types were assigned to the clusters based on the gene expression profiles.

#### Differential Expression Analysis

Differential expression testing between conditions within the same cell cluster was performed using the FindMarkers function, again using Wilcoxon rank-sum tests based on the normalized, but otherwise untransformed expression data.

## Results

### The Number of IL-5Rα+ Cells and Levels of IL-5 Are Elevated in Nasal Secretions of Aspirin-Exacerbated Respiratory Disease Patients

**Figure 1:**
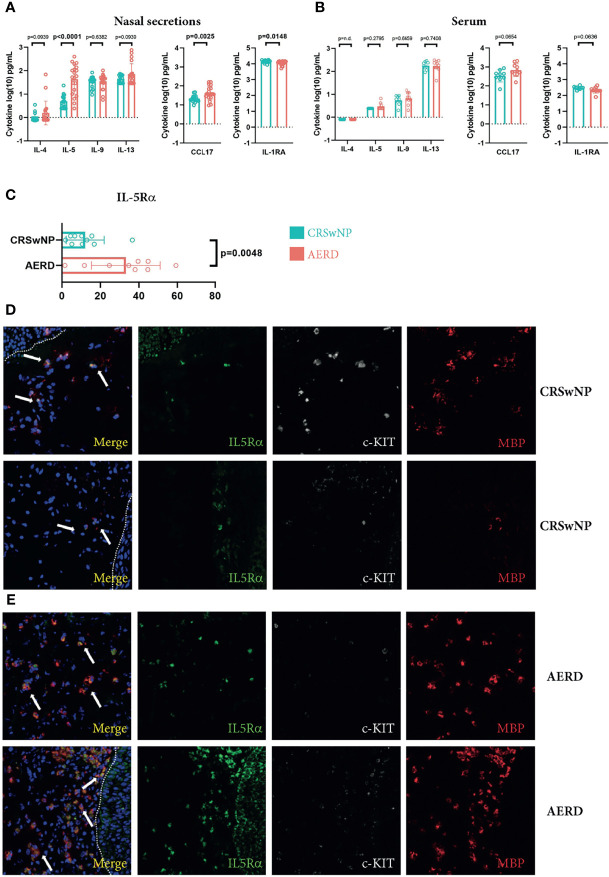
Increased IL-5 and IL-5Rα levels in patients suffering from AERD. **(A, B)** IL-4, IL-5, IL-9, IL-13, CCL17 and IL-1RA levels (y-axes, pg/ml) in **(A)** nasal secretions or **(B)** serum of patients suffering from CRSwNP (blue, n = 9) or AERD (red, n = 10) measured by MSD platform. **(C)** Quantification of the percentage of IL-5Rα cells of total cells (x-axes) in nasal polyp tissues of patients suffering from CRSwNP (blue, n = 9) or AERD (red, n = 10). **(D, E)** Representative immunofluorescence staining of IL-5Rα-positive cells (green, white arrows at merge) in polyp biopsies of **(D)** CRSwNP or **(E)** AERD patients. Tissues were co-stained with c-KIT (white) and MBP (red). Dotted lines indicate epithelium. Cytokine concentrations were log-transformed for analysis. Significance levels are indicated in figures, bars, and error bars represent means with SDs. AERD, aspirin-exacerbated respiratory disease; CRSwNP, chronic rhinosinusitis with nasal polyps; MBP, major basic protein; MSD, mesoscale discovery.

To elucidate inflammatory cytokine patterns distinguishing polyps from aspirin-intolerant versus tolerant patients, we selected 10 AERD and 9 CRSwNP patients and performed comprehensive profiling of nasal secretions and serum using the multiplex MSD platform (**Figure S3**). Patients’ profiles were well balanced in terms of age and allergic sensitization (**Table 1**). We found a significant increase in IL-5 (p < 0.0001) and a trend towards increased IL-13 levels (p = 0.0939) in the nasal secretions of AERD-affected patients (**Figure 1A**), as compared to patients with CRSwNP. In line, the T2-associated chemokine CCL17 showed higher levels in nasal secretions of AERD patients (p = 0.0025), while the IL-1 receptor antagonist (IL-1RA) was significantly elevated in nasal secretions of CRSwNP patients (p = 0.0148). These differences were evident in nasal secretions, but not in respective serum samples of the two patient groups (**Figure 1B**). Our observation that AERD patients had significantly elevated levels of IL-5 led us to investigate if this also resulted in the attraction of major IL-5-responsive cell populations such as eosinophils or MC (20). By subjecting polyp biopsies to immunofluorescence, we found that the percentage of IL-5Rα+ cells was significantly higher in AERD as compared to CRSwNP patients (**Figure 1C**) (p = 0.0048). Co-staining with c-KIT and MBP showed that a large number of IL-5Rα+ cells stained positive for MBP, indicating that the IL-5Rα-expressing cells largely belonged to the MC and/or eosinophil compartment (21) (**Figures 1D, E**). Given these findings and recent data on the pronounced T2 signature in AERD (22), we aimed to characterize disease-associated cell subsets in AERD and CRSwNP in more detail using scRNA-seq, potentially revealing differences also on a molecular level.

### Single-Cell RNA Sequencing Detects a Diverse Set of Cell Types in Human Polyps of Aspirin-Exacerbated Respiratory Disease and Chronic Rhinosinusitis With Nasal Polyps Patients

**Figure 2:**
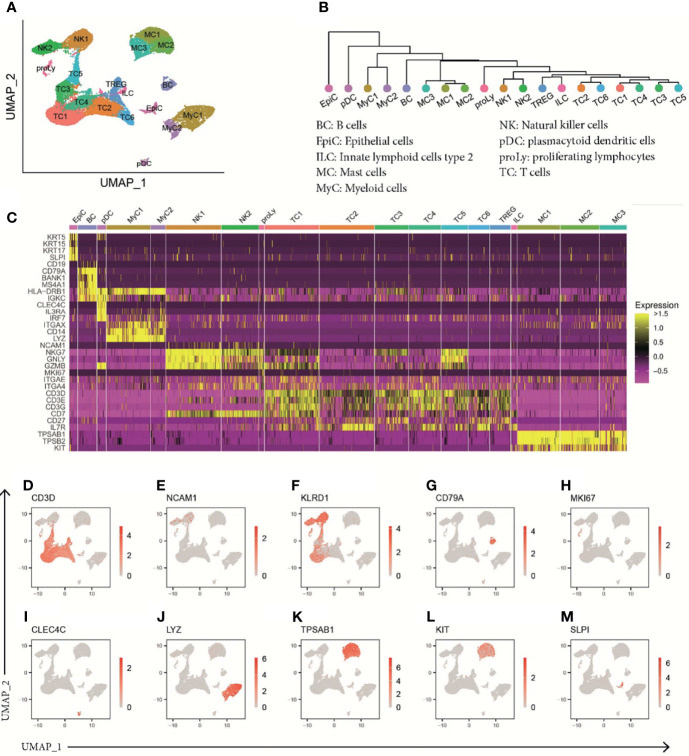
ScRNA-seq analysis of human polyp cells of patients with CRSwNP or AERD. **(A)** UMAP of unsupervised clustering of 32,358 polyp cells integrated from 19 donors (CRSwNP n = 9 and AERD n = 10) showing 19 differently color-coded clusters. **(B)** Phylogenetic tree showing relatedness of cell clusters based on the distance between the average gene signatures of each cluster using average linkage. **(C)** Heat map displaying z-score of selected markers of group identity. **(D–M)** Feature plots combining all samples showing expression of cluster-specific marker genes as overlaid onto UMAP plots. Red color displays intensity levels of natural log-transformed gene expression. UMAP, Uniform Manifold Approximation and Projection; scRNA-seq, single-cell RNA sequencing; CRSwNP, chronic rhinosinusitis with nasal polyps; AERD, aspirin-exacerbated respiratory disease.

To investigate differences in immune cell subsets in patients suffering from CRSwNP or AERD in detail, we performed scRNA-seq analysis of polyps using the 10x Genomics platform (14) (**Table S2** and **Figure S2**). To that aim, we pursued a flow cytometry sorting strategy specifically enriching for CD45+ cells. In total, we obtained 42,868 cells with a median of 2,073 cells per sample, a median of 4,084 mapped reads, and 1,485 expressed genes per cell (**Table S3**). This resulted in a total of 32,358 cells after quality control that were used for further bioinformatics analysis (**Table S4**). Unsupervised clustering of the cells revealed 19 different clusters showing good representation and similar distribution in all patients (**Figures 2A**, **S4**). To resolve individual cluster identities, we used a combination of canonical cell markers and the top 30 most upregulated genes per cluster (**Table S5**) as well as a hierarchical clustering tree of the distance between clusters (**Figure 2B**). The 19 identified clusters belonged to the following major cell subtypes as identified by signature genes (**Figures 2C–M**): seven T-cell clusters (*CD3D+*; TC1–TC6 and regulatory T cells (Treg); **Figures 2C, D**), two natural killer cell clusters (*NCAM-1+* and *KLRD1+*; NK1-2; **Figures 2C–F**), innate lymphoid cells type 2 (*IL7R+/CD3D−*; ILC2; **Figure 2C**), B cells (*CD79A+*; BC; **Figures 2C, G**), proliferating lymphocytes (*MKI67+*; proLy; **Figures 2C, H**), plasmacytoid dendritic cells (pDCs) (*CLEC4C+*; pDC; **Figures 2C, I**), two myeloid cell clusters (*LYZ+*; MyC1–2; **Figures 2C, J**), three MC clusters (*TPSAB1+*, *KIT*+; MC1–3; **Figures 2C, K, L**), and epithelial cells (*SLPI+*; EpiC; **Figure 2M**). In our further analysis, we focused on EpiC, MCs, TC, and MyC, as they showed the most prominent transcriptomic differences between the disease subtypes (**Table S6**).

### Mast Cells of Aspirin-Exacerbated Respiratory Disease Patients Are Characterized by Increased Expression of T2 Cytokines, VEGFA, and CSF1

**Figure 3:**
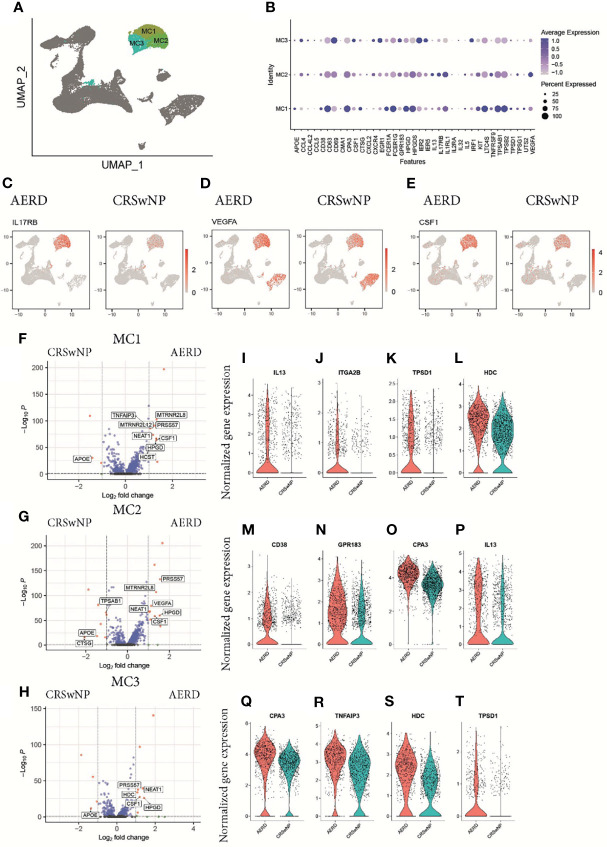
Characterization of mast cells in patients with AERD or CRSwNP. **(A)** Color-coded UMAP plot of scRNA-seq MC subclusters. **(B)** Dot plots showing selected marker genes (x-axis) within three mast cell populations (MC1–MC3) displaying the z-score of the gene average expression across the three clusters (blue color) and frequency of gene expression (circle size). Feature plots showing natural log-transformed gene expression of **(C)** IL17RB, **(D)** VEGFA, and **(E)** CSF1 in patients with AERD (left) or CRSwNP (right). **(F–H)** Volcano plots displaying differentially regulated genes in AERD versus CRSwNP in **(F)** MC1, **(G)** MC2, and **(H)** MC3. Genes with a Bonferroni-corrected p-value <0.05 and a log-fold change >1 are labeled in red. **(I–T)** Violin plots show natural log-transformed normalized gene expression for selected DEG in AERD (red) versus CRSwNP (blue) patients. AERD, aspirin-exacerbated respiratory disease; CRSwNP, chronic rhinosinusitis with nasal polyps; DEG, differentially expressed genes; MC, mast cell; FDR, false discovery rate; UMAP, Uniform Manifold Approximation and Projection; scRNA-seq, single-cell RNA sequencing.

Transcriptomic profiling identified three clusters of tryptase+ (*TPSAB1*) and carboxypeptidase A3+ (*CPA3*) MC (MC1–MC3) (**Figures 3A, B**). Differential gene expression analysis between these MC clusters revealed unique transcriptomic features enabling them to exert subset-specific functions (10). MC1 had higher expression levels of chymase (*CMA1*) and cathepsin G (*CTSG*) as compared to the other two clusters, indicating that this population is most consistent with the recently described *TPSAB1*+ and *CMA1+* subepithelial MC subset (MC_TC_) (**Figure 3B**) (23, 24). The MC2 and MC3 clusters showed lower expression of CD117 (*KIT*) and the high-affinity IgE receptor FcϵR1α (*FCER1A*) and, thus, correspond most likely to an intraepithelial tryptase-expressing MC subset (MC_T_) (24) (**Figure 3B**). Specific for MC2 cells, we detected an enhanced expression of *IL5* and *IL13*, the receptors for IL-33 (interleukin 1 receptor-like 1, *IL1RL1*) and IL-25 (interleukin 17 receptor B, *IL17RB*), and vascular endothelial growth factor A (*VEGFA*) (**Figure 3B**), indicating that this cluster not only is poised towards responding to the epithelial-derived alarmins IL-33 and IL-25 but also has the capacity to amplify T2 proinflammatory responses. MC3 cells were characterized by higher levels of the chemokine ligands *CCL4* and *CCL5* known to be involved in enhancing allergic inflammation by inducing an influx of eosinophils, basophils, and macrophages to mucosal sites (25) as well as growth factors [colony-stimulating factor 1 (*CSF1*) and epidermal growth factor 1 (*EGFR1*)] as compared to MC1 and MC2 (**Figure 3B**).

In patients suffering from AERD as compared to CRSwNP, the top upregulated differentially expressed genes (DEG) across all MC clusters included *IL17RB* (**Figure 3C**), *VEGFA* (**Figure 3D**), *CSF1* (**Figure 3E**), the nuclear enriched abundant transcript *NEAT1*, which is thought to play a role in inflammasome activation (26), and the 15-hydroxyprostaglandin dehydrogenase *HPGD* (**Figures 3F–H**) (8). AERD-derived MC1 cells also showed significant higher *IL13* expression (**Figures 3I**, **S5A**), a trend towards elevated *IL4* levels (**Figure S5B**), and expressed higher levels of the integrin alpha 2B (*ITGA2B*) (**Figure 3J**) as well as significantly higher levels of tryptase D (*TPSD1*, **Figure 3K**) and histamine decarboxylase (*HDC*; **Figure 3L**). The intraepithelial AERD-derived MC2 cluster had significantly increased expression of *CD38* (**Figure 3M**), *GP138* (**Figure 3N**), *CPA3* (**Figure 3O**), and *IL13* (**Figure 3P**), indicating that it belongs to a previously identified MC_T_ subset especially involved in respiratory tract inflammation (10). MC3 in AERD patients showed significantly increased expression of *CPA3* (**Figure 3Q**) and the TNF-α induced protein 3 *TNFAIP3* (**Figure 3R**). Polymorphism in the latter gene has been associated with chronic rhinosinusitis pathogenesis (27). Similar to MC1, MC3 demonstrated upregulation of *HDC* (**Figure 3S**) and tryptase D (*TPSD1*, **Figure 3T**). Interestingly, apolipoprotein E (*ApoE*), which has previously been attributed a protective role in respiratory disease (28), was strongly and significantly downregulated across all MC clusters in AERD patients (**Figures 3F–H**).

### Increased Expression of Genes Associated With T2 Immune Response and Tissue Remodeling in Epithelial Cells Derived From Aspirin-Exacerbated Respiratory Disease Patients

**Figure 4:**
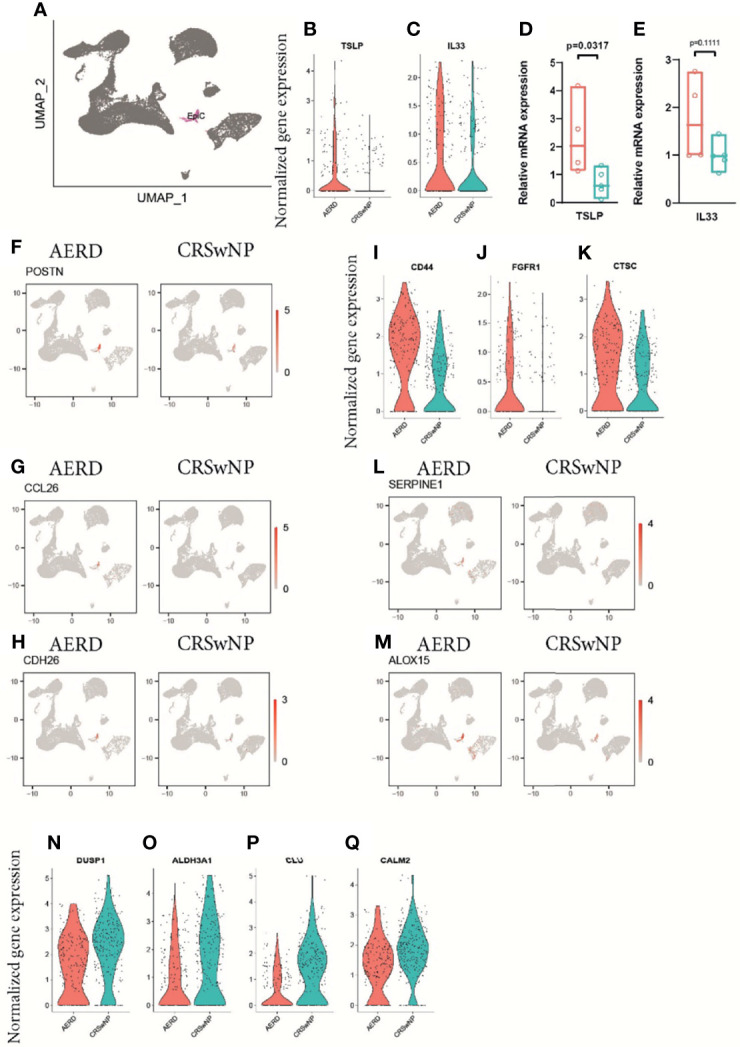
Gene expression of epithelial cells of patients with AERD or CRSwNP. **(A)** Color-coded UMAP plot of scRNA-seq epithelial cell cluster. **(B, C)** Violin plots showing natural log-transformed normalized gene expression for **(B)** *TSLP* and **(C)** *IL33* in AERD (red) versus CRSwNP (blue) patients. **(D, E)** Quantitative real-time PCR of bulk nasal tissue isolates for **(D)** *TSLP* and **(E)** *IL33* confirming scRNA-seq data. Line represents median values, and p-values are indicated in plots. **(F–H)** Feature plots identifying **(F)** *POSTN*, **(G)** *CCL26*, and **(H)** *CDH26* in patients with AERD (left) and CRSwNP (right). **(I–K)** violin plots showing natural log-transformed normalized gene expression for **(I)** *CD44*, **(J)** *FGFR1*, and **(K)** *CTSC* in AERD (red) versus CRSwNP (blue) patients. **(L, M)** Feature plots identifying **(L)** *SERPINE1* and **(M)** *ALOX15* in patients with AERD (left) and CRSwNP (right). **(N–Q)** Violin plots showing natural log-transformed normalized gene expression for DEG downregulated in AERD (red) versus CRSwNP (blue) patients. Feature plots: red color displays intensity levels of natural log-transformed gene expression. AERD, aspirin-exacerbated respiratory disease; CRSwNP, chronic rhinosinusitis with nasal polyps; DEG, differentially expressed genes; UMAP, Uniform Manifold Approximation and Projection; scRNA-seq, single-cell RNA sequencing.

Comparing the epithelial cell cluster in AERD and CRSwNP patients (**Figure 4A**), we observed a trend towards, but not a significant, increase in the T2-associated alarmins thymic stromal lymphopoietin *TSLP* and *IL33* in AERD using scRNA analysis (**Figures 4B, C**). Further investigations using bulk tissue RT-PCR showed significantly increased TSLP and a trend towards enhanced IL33 expression in AERD as compared to CRSwNP (**Figures 4D, E**). Corroborating our immunofluorescence data of a more pronounced eosinophilic infiltration in AERD associated with T2 inflammation, genes promoting eosinophil recruitment such as periostin (*POSTN*) (**Figure 4F**), *CCL26* (29) (**Figure 4G**), and cadherin 26 (*CDH26*) (**Figure 4H**) were significantly higher expressed. Among DEG promoting cell proliferation and tissue remodeling, genes such as Wnt pathway-associated *CD44* (9) (**Figure 4I**) and fibroblast growth factor receptor 1 (*FGFR1*) (30) (**Figure 4J**) were upregulated in AERD. The dipeptidyl peptidase 1 (*CTSC*) (**Figure 4K**), a ubiquitously expressed gene involved in the activation of neutrophil serine protease, elastase, and cathepsin G (31), also showed significant elevation in AERD. Likewise, the serine protease inhibitor *SERPINE1* (**Figure 4L**), previously observed to be elevated in epithelial cells of CRSwNP patients (32), proved to be upregulated in AERD similar to the arachidonic acid pathway-associated gene *ALOX15* (8) (**Figure 4M**). Among the top downregulated DEG in AERD, we observed the protein phosphatase *DUSP1* (**Figure 4N**), which has also been implicated to play a role in modulating T2 responses: knockdown of *DUSP1* in nasal epithelial cells induces a strong upregulation of *TSLP* expression and production (33). Expression of the carbohydrate metabolism-associated gene *ALDH3A1* (**Figure 4O**), the antimicrobial protein clusterin (*CLU*) (**Figure 4P**), and calmodulin 2 (*CALM2*) (**Figure 4Q**), a calcium-binding protein involved in mediating ciliary beat responses in the human nasal epithelium (34), were also significantly reduced in cells from AERD as compared to CRSwNP patients.

### Mature Myeloid Dendritic Cells Support T2 Inflammation in Aspirin-Exacerbated Respiratory Disease

**Figure 5:**
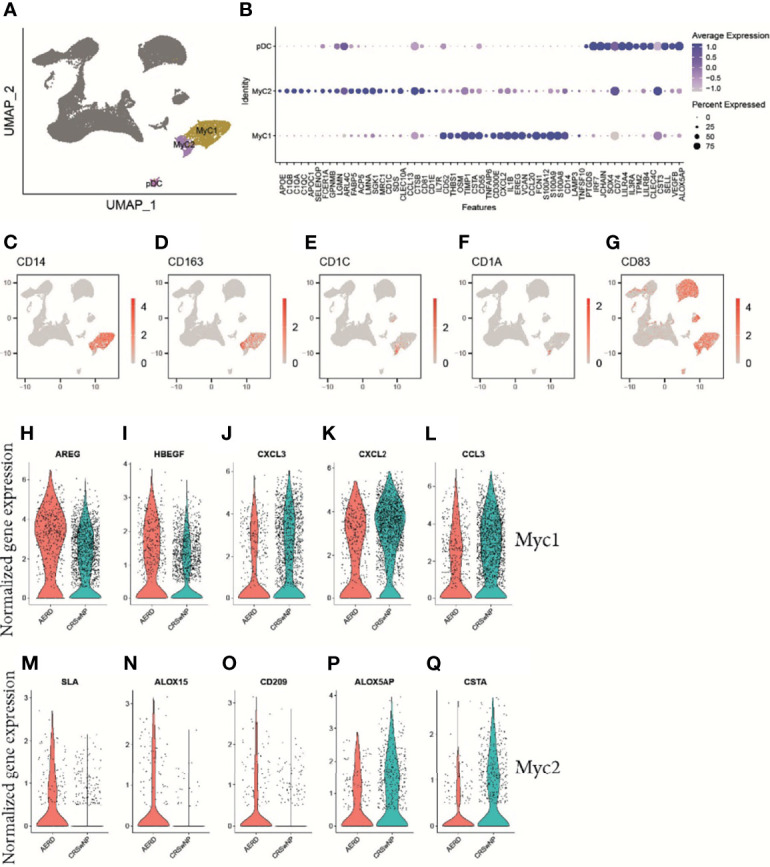
Gene expression of myeloid cells and plasmacytoid dendritic cells in patients with AERD or CRSwNP. **(A)** Color-coded UMAP plot of scRNA-seq MyC and pDC subclusters. **(B)** Dot plots showing selected marker genes within MyC1, MyC2, and pDC displaying z-score of average expression (blue color intensity) and frequency of gene expression (circle size). **(C–G)** Feature plots identifying combined natural log-transformed normalized expression. Red color displays intensity levels of natural log-transformed gene expression. **(H–Q)** Violin plots showing natural log-transformed normalized gene expression for selected DEG in AERD (red) versus CRSwNP (blue) patients in **(H–L)** MyC1 and **(M–Q)** MyC2. AERD, aspirin-exacerbated respiratory disease; CRSwNP, chronic rhinosinusitis with nasal polyps; DEG, differentially expressed genes; MyC, myeloid cell; pDC, plasmacytoid dendritic cell; UMAP, Uniform Manifold Approximation and Projection.

When characterizing myeloid cells, MyC1 constituted the largest cell cluster harboring a subset of *CD14+*, *CD163+*, and lysozyme (*LYZ*+) macrophages (**Figures 5A–D**). We further identified the antimicrobial peptides *S100A8* and *S100A9*, the proinflammatory molecules *IL1B* and *CXCL2*, the EGRF ligand *EREG*, and the CCR6 ligand *CCL20* among the top gene markers in this cluster (**Figure 5B**). The rather small MyC2 cluster mainly comprised DCs as indicated by the expression of the DC-specific markers *CD1c* and *CD1a* (**Figures 5A, B, E, F**). While both MyC clusters were important producers of *TNF* and *IL10* (**Figures S5C, D**), Myc2 cells were the only cells expressing chemokines associated with T2 immunity, namely, *CCL17* and *CCL22* (**Figures S5E, F**). When analyzing the maturation status of MyC/pDC subsets, we saw a strong expression of the maturation marker *CD83* in all subsets (**Figure 5G**). We also found a very small population of pDC-expressing characteristic markers such as *CLEC4C* (BDCA-2), *LILRA4*, *LILRB4*, *IL3RA* (CD123), and *IRF7* (**Figures 5A, B**). Specific differences between AERD and CRSwNP in MyC1 cells revealed an upregulation of the T2-associated amphiregulin (*AREG*) and its paralogue heparin-binding EGF-like growth factor (*HBEGF*) in AERD (**Figures 5H, I**), whereas *CXCL3*, *CXCL2*, and *CCL3*, all chemokines associated with type 1 immunity, were significantly downregulated (**Figures 5J, K, L**). The MyC2 cluster in AERD demonstrated an upregulation of *SLA*, an adapter protein, which negatively regulates TCR signaling, the arachidonate 15-LO (*ALOX15*), and DC-SIGN (*CD209*) (**Figures 5M–O**), as well as a downregulation of FLAP (*ALOX5AP*) and cystatin A (*CSTA*) (**Figures 5P, Q**).

### Characterization of T-Cell Subsets Reveals a Predominance of Cytotoxic Tissue-Resident Memory T Cells in Nasal Polyps in Aspirin-Exacerbated Respiratory Disease and Chronic Rhinosinusitis With Nasal Polyps Patients

**Figure 6:**
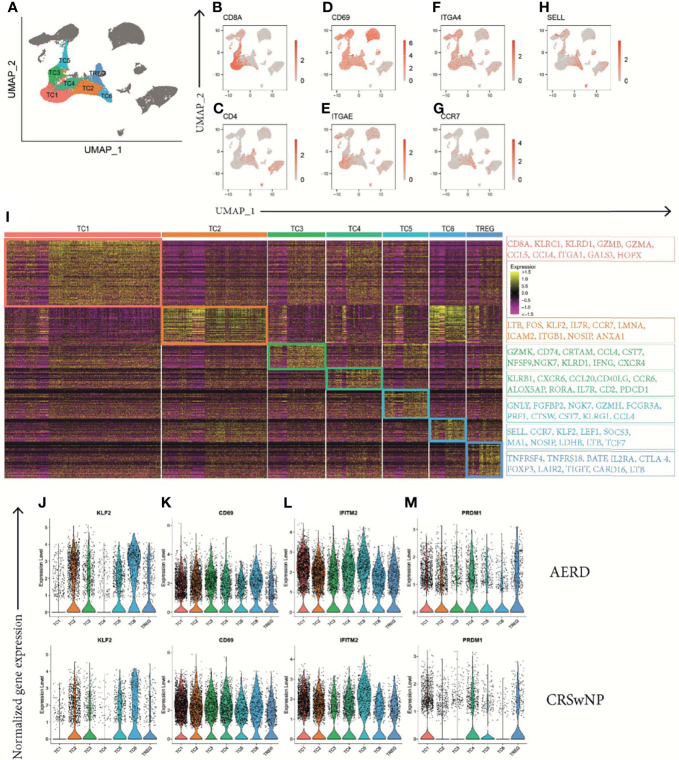
Identification of T-cell clusters in patients with AERD and CRSwNP. **(A)** Color-coded UMAP plot showing seven scRNA-seq TC subclusters with **(B–H)** corresponding feature plots identifying combined natural log-transformed normalized expression of **(B)** *CD8A*, **(C)** *CD4*, **(D)** *CD69*, **(E)** *ITGAE*, **(F)** *ITGA4*, **(G)** *CCR7*, and **(H)** *SELL*. Red color displays intensity levels of natural log-transformed gene expression. **(I)** Heat map showing z-score of the expression across clusters with selected top color-coded DEG highlighted in boxes characterizing TC cluster identity. **(J–M)** Color-coded violin plots showing natural log-transformed normalized gene expression for selected DEG in all TC clusters of (top) AERD as compared to (bottom) CRSwNP. AERD, aspirin-exacerbated respiratory disease; CRSwNP, chronic rhinosinusitis with nasal polyps; TC, T cell; DEG, differentially expressed genes; UMAP, Uniform Manifold Approximation and Projection; scRNA-seq, single-cell RNA sequencing.

The CD3D+ T-cell cluster contained 7 subclusters (TC1–TC6 and Treg, **Figure 6A**) with an αβ T-cell receptor (TCR) phenotype (*TRAC+*) (**Figure S6A**) and mutually exclusive populations of *CD8A*+ (**Figure 6B**) and *CD4*+ (**Figure 6C**) T cells. The majority of *CD3D+* T cells were identified as tissue-resident memory T-cells (T_RM_) based on *CD69* and integrin subunit alpha E *ITGAE* (CD103) or integrin subunit alpha 4 *ITGA4* (CD49d) expression (**Figures 6D–F**) (35, 36). Interestingly, two subsets (TC2 and TC6) showed upregulation of chemokine receptor 7 (*CCR7*) and/or L-selectin *SELL* (CD62L) while simultaneously expressing *CD69* (**Figures 6G, H**). When analyzing DEG within the T-cell clusters, the largest cluster (TC1) displayed features of cytotoxic *CD8A*+ T_RM_ cells with a type 1 (*IFNG*) phenotype (**Figures 6I**, **S6B**), a significant upregulation of the cytotoxic molecule granzyme B (*GZMB*) (**Figure S6C**), and the immune inhibitory receptor killer cells lectin-like receptor C1 (*KLRC1*) and D1 (*KLRD1*) (**Figure 6I**). The TC2 cluster mainly displayed a *CD4+* helper T-cell phenotype with elevated expression levels of lymphotoxin beta (*LTB*) (**Figures 6I**, **S6D**), the member of the zinc finger transcription family *Kruppel-like factor-2* (*KLF2*) (**Figure S6E**), and the proto-oncogene *FOS* (**Figure 6I**). As upregulation of *KLF2* has previously been associated with regulating the maintenance of quiescence in T cells (37) and we could not identify a specific cytokine signature in this cluster, we hypothesize that TC2 cells are most consistent with memory T cells in a resting state (38). The phenotype of the smaller TC3 and TC5 clusters phenotypically corresponds to terminally differentiated *CD8A*+ cytotoxic effector memory cells as indicated by their unique expression of killer cell lectin-like receptor G1 (*KLRG1*) (**Figures 6I**, **S6F**), paralleled by significant downregulation of CD127 (*IL7R*) (**Figure S6G**) (39) and other genes such as granulysin (*GNLY*) or natural killer cell granule protein 7 (*NKG7*) that further endow these T cells with a striking cytotoxic potential (**Figure 6I**). The TC4 cluster comprised both *CD4+* and *CD8A+* cells and was further characterized by differential expression of CD161 (*KLRB1*) (**Figures 6I**, **S6H**), *CXCR6*, *CCL20*, and *CCR6* (**Figure 6I**), a phenotype most consistent with recently identified mucosal-associated invariant T cells (11). These cells also express the Th17-related transcription factor *RORA* (**Figure 6I**) (40), *IL17A* (**Figure S6I**), and *IL26* (**Figure S6J**). Finally, the smallest T-cell clusters, TC6 and Treg, were phenotypically characterized as *CD4+* T cells. TC6 displayed a central memory (T_CM_) phenotype (*CCR7+*, *SELL+*, *KLF2+*, *TCF7+*, *ITGAE−*, and *ITGA4−*) (**Figure 6I**), while Treg expressed *FOXP3* (**Figure S6K**), CD25 (*IL2RA*), and *CTLA-4* (**Figure 6I**).

### Gene Expression of T-Cell Subsets in Aspirin-Exacerbated Respiratory Disease Supports Skewing of T2 Immune Responses

In polyp tissue of AERD as compared to CRSwNP, the transcription factor *KLF2* was upregulated in TC2, 3, 4, and 6 (**Figure 6J** and **Table S6**). KLF2 is rapidly lost after TCR-mediated signaling or upon cytokine-induced T-cell activation, indicating a more resting state of T cells in AERD. This finding was supported by the downregulation of type II C-lectin receptor *CD69*, a classical early marker of T-cell activation, in almost all AERD-derived TC clusters excluding TC3 (**Figure 6K** and **Table S6**). Strikingly, we observed a strong upregulation of the interferon-inducible transmembrane gene 2 (*IFITM2*) in all T-cell subsets of AERD (**Figure 6L** and **Table S6**). The IFITM family not only plays a major role in conferring cellular resistance to viruses, but recent evidence suggests that *IFITM2* primarily polarizes T-cell immunity towards a T2 immune response and simultaneously acts as a negative regulator of type 1 differentiation (41). A protein with comparable capacities in skewing T2 immunity and limiting type 1 and type 17 differentiation is the B lymphocyte-induced maturation protein-1 (*PRDM1*) (42), whereas *PRDM1* expression in CRSwNP was restricted to TC1, 4, 5, and Treg; we found additional upregulation in the Th2 clusters TC2 and TC6 in AERD (**Figure 6M** and **Table S6**).

### Cell Subsets in Aspirin-Exacerbated Respiratory Disease Show Upregulation of Genes Related to Stress Response and Activation of T2 Immunity

**Figure 7:**
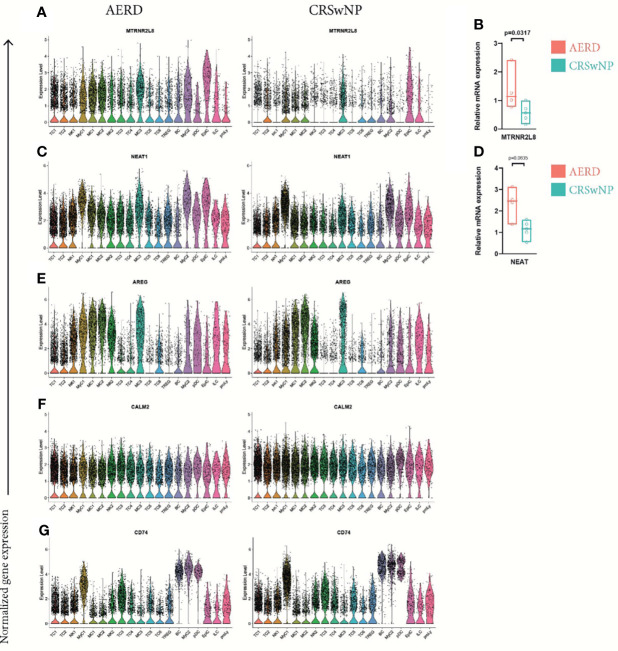
Differentially expressed genes comparing all cell clusters of AERD and CRSwNP. Color-coded violin plots showing natural log-transformed normalized gene expression for **(A)** *MTRNR2L8*, **(C)** *NEAT1*, **(E)** *AREG*, **(F)** *CALM2*, and **(G)** *CD74* in all cell clusters of (left) AERD as compared to (right) CRSwNP. Quantitative real-time PCR of bulk nasal tissue isolates for **(B)** *MTRNR2L8* and **(D)** *NEAT1* confirming scRNA-seq data. Line represents median values, and p-values are indicated in plots. AERD, aspirin-exacerbated respiratory disease; CRSwNP, chronic rhinosinusitis with nasal polyps.

When further investigating disease-specific differences, we found that the mitochondrial-derived peptides humanin-like 8 (*MTRNR2L8*) (**Figure 7A**) and humanin-like 12 (*MTRNR2L12*) (**Figure S7A**) ranged within the top 35 upregulated DEG in 15 of 19 cell clusters in AERD excluding only TC2, TC6, ILC, and proliferating lymphocytes. Induced by oxidative stress, humanins have the capacity to exert broad cytoprotective effects (43). We corroborated our scRNA-seq by performing bulk tissue quantitative real-time PCR of *MTRNR2L8* from whole biopsies of the same patient groups (**Figure 7B**). To rule out the potential role of corticosteroids in inducing these mitochondrial stress-related genes (44), we analyzed *MTRNR2L8* and *MTRNR2L12* in each patient. Of note, also some CRSwNP patients devoid of topical corticosteroid therapy showed an elevation of both genes (**Figures S7B, C**).

We further identified the nuclear enriched abundant transcript 1 (*NEAT1*), a long non-coding RNA previously reported to play a role in promoting Th17 and Th2 immunity, to be significantly upregulated in most leukocyte subsets of AERD as compared to CRSwNP (**Figure 7C**) (45, 46). RT-PCR of *NEAT1* in bulk tissue confirmed our results although not reaching significance (**Figure 7D**). Known to be associated with amplifying T2 immunity, *AREG* was significantly increased in TC1, TC2, NK1, NK2, and MyC1 cell clusters of AERD as compared to CRSwNP, with comparable expression in the remaining cell subsets (**Figure 7E**). Downregulation of *CALM2* was observed not only in EpiC but also in 16 out of 19 clusters in AERD as compared to CRSwNP (**Figure 7F**). *CD74*, an important ligand of the cytokine macrophage-inhibitory factor (*MIF*) and involved in numerous signaling pathways, ranged among the top 30 downregulated genes within BC, MyC1, NK2, pDC, TC4, TC5, and Treg leukocyte clusters in AERD (**Figure 7G**).

## Discussion

This is the first report providing a comprehensive characterization of epithelial cells, MC, myeloid cells, as well as T-cell subsets in nasal polyp tissue of patients suffering from AERD as compared to CRSwNP patients using a scRNA-seq approach. Importantly, we minimized the potential bias of atopy in our dataset by employing equal distribution of allergic patients in both disease groups. We revealed that i) nasal secretions of AERD patients display significantly increased levels of T2 molecules such as IL-5 and CCL17; ii) AERD-derived epithelial and MC clusters show a T2-associated inflammatory phenotype; iii) epithelial cells and MC complement one another in terms of gene expression associated with the 15-LO pathway in AERD patients, and cytotoxic T cells comprise a large part of infiltrating T cells; iv) stress-associated genes are upregulated in the majority of AERD-derived immune cell subsets.

Dysregulation of the arachidonic acid pathways plays an important role in the pathogenesis of AERD resulting in hypersensitivity to anti-inflammatory drugs inhibiting the cyclooxygenase pathway as a hallmark of this disease. We detected genes associated with the 15-LO pathway to be highly elevated in patients suffering from AERD as compared to CRSwNP patients: *ALOX15* encoding the 15-LO in epithelial cells as well as in MyC2 DCs and, of note, the further downstream acting *HPGD* in all three MC clusters, whereas previous data described elevated levels of *ALOX15* in epithelial cells of AERD patients as compared to CRSwNP patients, differential expression of *HPGD* in MC had not been detected before (8). Importantly, our findings not only corroborate but also complement the recently proposed hypothesis of epithelial and MC transmetabolism in AERD: epithelial cells generate 15-HETE mediated by 15-LO and then pass the metabolite to closely located MC for oxidation into 15-oxo-ETE by HPGD, the end product of the 15-LO pathway (47). The clinical importance of this pathway in AERD is supported by observations that patients who benefit from aspirin desensitization therapy show pronounced levels of 15-HETE baseline plasma levels (48) and high HPGD expression in sputum cells (49).

AERD is a disease associated with enhanced T2 inflammation and eosinophilia (50); however, the interplay of the different immune cell subsets to establish a T2 environment is still not clear. Here, we observed not only overall elevated levels of IL-5 and CCL17 in nasal secretions but also increased expression of alarmins (*TSLP* and *IL33*) as well as the eosinophil attractants *POSTN* (51), *CDH26* (52), and *CCL26* (29) in epithelial cells of AERD patients. This finding was complemented by significantly elevated levels of the alarmin receptors IL25R (*IL17RB*) and IL33R (*IL1RL1*) in all MC clusters as well as *IL13* in MC1 and MC2 of AERD patients. Evidence from previous data shows that IL-13 stimulation induces 15-LO expression in epithelial cells resulting in CCL26 production (53). It is, thus, conceivable that the interplay of upregulated *IL13* levels in MC drives the 15-LO-mediated expression of *CCL26* and *POSTN* in epithelial cells resulting in eosinophil recruitment (53, 54). MC cluster MC2 in AERD also showed signs of a recently described inflammatory CD117^high^ CD38^high^ subepithelial MC_T_ phenotype, which is specifically expanded during T2 inflammation (10). The significant expression of T2-associated molecules in MC and epithelial cells allowing their tight interaction renders them potential key players in promoting AERD.

The accentuated T2 pattern in AERD was also supported by increased expression of signature genes such as *PRDM1* (42) and *IFITM2* (41) in T cells as well as *AREG* in MyC, NK, and T-cell clusters derived from AERD patients. Recent data demonstrated that epithelial cell-derived IL-33 induced production of AREG in Th2 cells leading to enhanced eosinophil activation and expression of osteopontin, which might be involved in lung fibrosis (55). It is conceivable that a similar mechanism, involving AREG production not only in T cells but also in macrophages and NK cells, might be operative in the pathogenesis of AERD. Importantly, nasal polyp-derived T-cell clusters not only demonstrated a strong cytotoxic potential but partly also exhibited a quiescent phenotype that was more pronounced in AERD-derived T cells as marked by increased *KLF2* (37), decreased *CD69*, and limited cytokine expression.

Interestingly, we observed across all clusters an upregulation of genes associated with cellular stress response, namely, *NEAT1* and humanin-encoding genes *MTRNR2L8* and *MTRNR2L12* in AERD as compared to CRSwNP patients. In this respect, the proinflammatory molecule NEAT1 has been reported to promote the assembly of the inflammasome (26) as well as T2 inflammatory responses (45). The recent observation that NEAT1 is significantly higher expressed in patients suffering from severe as compared to mild COVID-19 also supports its important proinflammatory role (56). Humanins have been reported to be upregulated predominantly upon oxidative stress in inflammatory diseases and to have a protective effect in early atherosclerosis (43) and cardiovascular disease (57). One potential explanation for our findings is that there is increased cellular stress in cells of AERD due to the higher production of eosinophil-derived reactive oxygen species in these patients. This not only may result in inflammasome activation by NEAT1 upregulation but also may at the same time lead to increased humanin production in an attempt to protect the cells from damage and stress-related excessive immune responses (58).

We are aware that this study has some limitations. First, all were AERD patients, but only 55% of patients with CRSwNP were under current nasal corticosteroid therapy at the time of the study. However, as we observed increased inflammatory and proliferative signs in the group of AERD patients despite all of them undergoing topical corticosteroid therapy, we are confident that this has only limited impact on our results. Second, in line with previous observations, eosinophils could not be mapped in the scRNA-seq approach (8, 9). This phenomenon can most likely be explained by the damaging effect of tissue processing on eosinophils or by the low abundance of RNA in eosinophils, rendering them difficult to be detected. Third, the relative scRNA-seq output of epithelial cells was lower as compared to immune cell subsets, as epithelial cells are more likely to undergo cell death upon tissue processing. Fourth, due to the predominance of women with AERD and to our emphasis on balancing allergic and non-allergic patients with both disease entities, our disease groups are not well-balanced in terms of sex. While we cannot exclude a certain sex bias in our dataset, our main results are in line with previously published observations (8).

In summary, we provide a thorough and detailed phenotypic analysis of various immune subsets involved in driving AERD by using nasal secretions as well as scRNA-seq. Our findings indicate a pivotal role of the interplay between epithelial and MC in promoting not only gene expression associated with the 15-LO pathway but also T2 inflammation and eosinophil recruitment in AERD. Interestingly, we observed cellular stress responses across all immune cell subsets that warrant further investigation about the potential role of these genes in driving AERD. Our findings contribute to a more detailed and integrative understanding of the pathogenesis of AERD and may help to identify novel treatment targets for this difficult-to-treat disease.

## Data Availability Statement

The datasets presented in this study can be found in online repositories. The names of the repository/repositories and accession number(s) can be found https://www.ncbi.nlm.nih.gov/geo/, GSE196169.

## Ethics Statement

The studies involving human participants were reviewed and approved by the Ethics committee of the Medical University of Vienna (EK No. 1956/2018), Vienna, Austria. The patients/participants provided their written informed consent to participate in this study.

## Author Contributions

CBa, SVM, SS, and JE-D designed the study. CBa, SS, TB, TQ, VN-L contributed to patient recruitment and sampling. CBa, SVM, WB, TK, VS, NJC and GR performed the experiments. TK, CBo, CBa, MF, SVM, and JE-D performed the data analysis. CBa, SVM, MF, JE-D, and SS wrote the manuscript. All authors critically revisedthe manuscript.

## Funding

This work was funded by AstraZeneca externally sponsored research grant No. NCR 18-14168. The funder was not involved in the study design, collection, analysis, interpretation of data, the writing of this article, or the decision to submit it for publication. MF is supported by a network grant of the European Commission (H2020-MSC-ITN-765104-MATURE-NK) to Arndt von Haeseler. SM and NC were supported by the FWF DK W 1248-B30 and SFB F4613 from the Austrian Science Fund (FWF).

## Conflict of Interest

The authors declare that the research was conducted in the absence of any commercial or financial relationships that could be construed as a potential conflict of interest.

## Publisher’s Note

All claims expressed in this article are solely those of the authors and do not necessarily represent those of their affiliated organizations, or those of the publisher, the editors and the reviewers. Any product that may be evaluated in this article, or claim that may be made by its manufacturer, is not guaranteed or endorsed by the publisher.

## Abbreviations

AERD, aspirin-exacerbated respiratory disease; CRSwNP, chronic rhinosinusitis with nasal polyps; DEG, differentially expressed genes; T2, type 2; 15-LO, 15-lipoxygenase; scRNA-seq, single-cell RNA sequencing; MC, mast cell; MSD, mesoscale discovery.
