# Supplementary material for: Comprehensive Analysis of Nasal Polyps Reveals a More Pronounced Type 2 Transcriptomic Profile of Epithelial Cells and Mast Cells in Aspirin-Exacerbated Respiratory Disease
Source: Front Immunol. 2022 Mar 28;13:850494. doi: 10.3389/fimmu.2022.850494 (PMC8996080; doi:10.3389/fimmu.2022.850494)
Supplement: Supplementary file 1 [file DataSheet_1.zip › Supplementary Material/Supplementary Figures 1-7.docx]

**Supplementary Figures S1-7:**

**
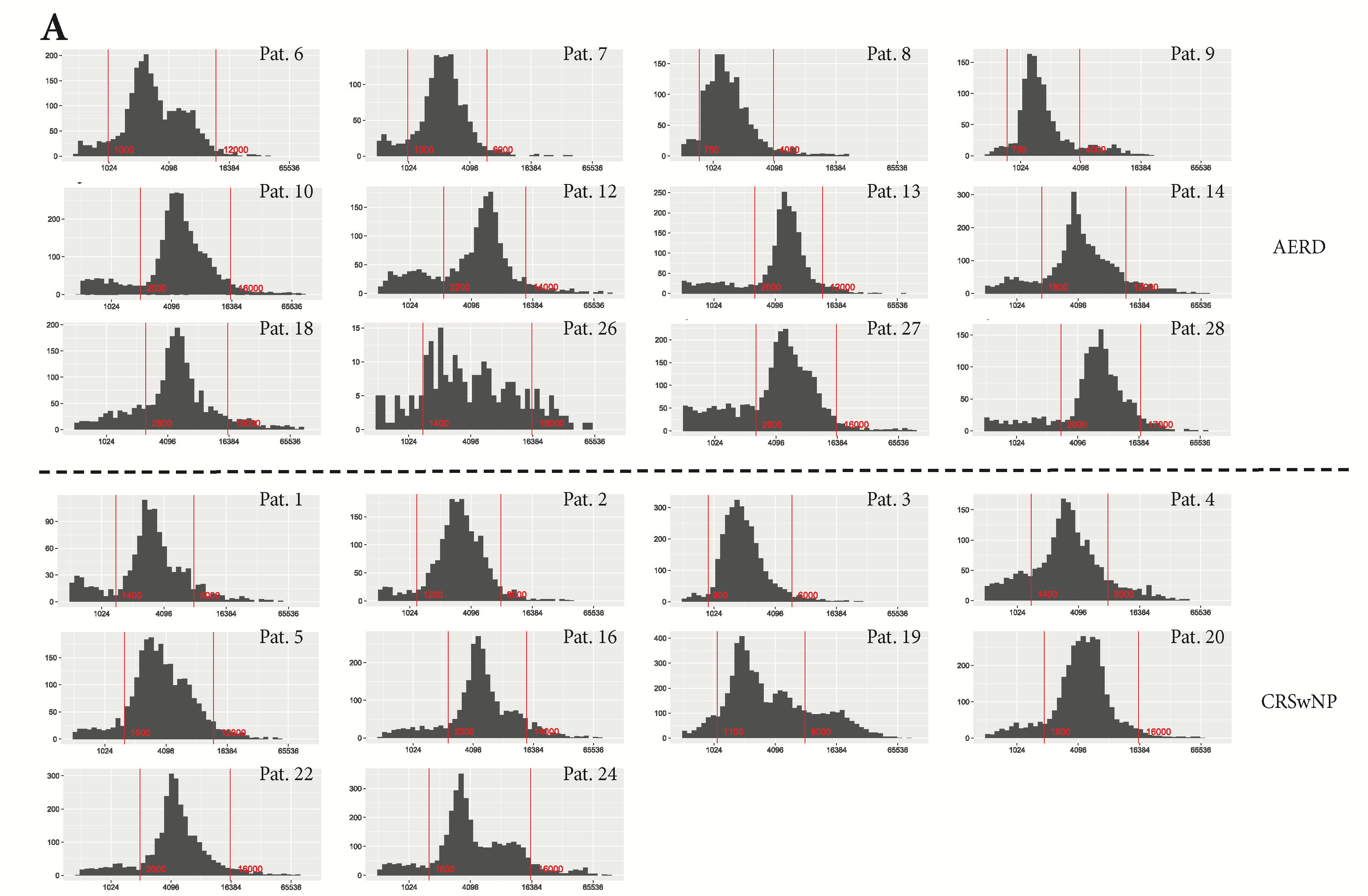
**

**
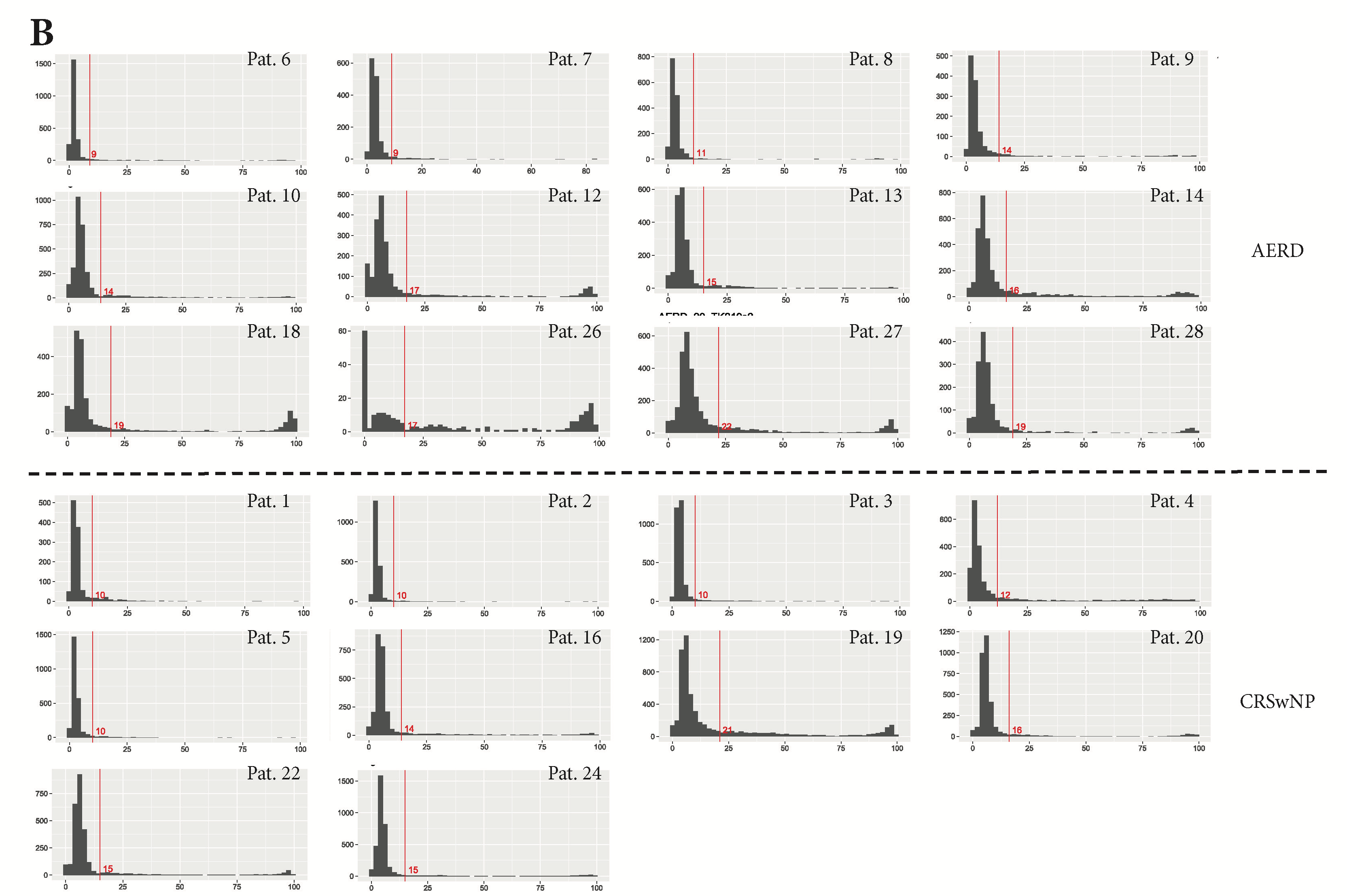
**

**Figure S1:** Histogram of A) count-depth per cell-barcode, for each patient and B) percentage of mitochondrial reads per cell-barcode. Red vertical lines and numbers show the chosen cutoff-values during quality control.

**Figure S2: Work flow chart** showing tools used for analyzing serum, nasal secretions and nasal polyp biopsies of patients suffering from AERD (n=10) or CRSwNP (n=9). Cytokine profiles in serum and nasal secretions were assessed using a multiplex mesoscale discovery U-plex approach. Biopsies of nasal polyps were subjected to immunofluorescence and transcriptome profiling using scRNA-seq (10x Genomics platform). Cells used for scRNA-seq were enriched for various immune subsets by flow cytometry sorting (Table E2).

**
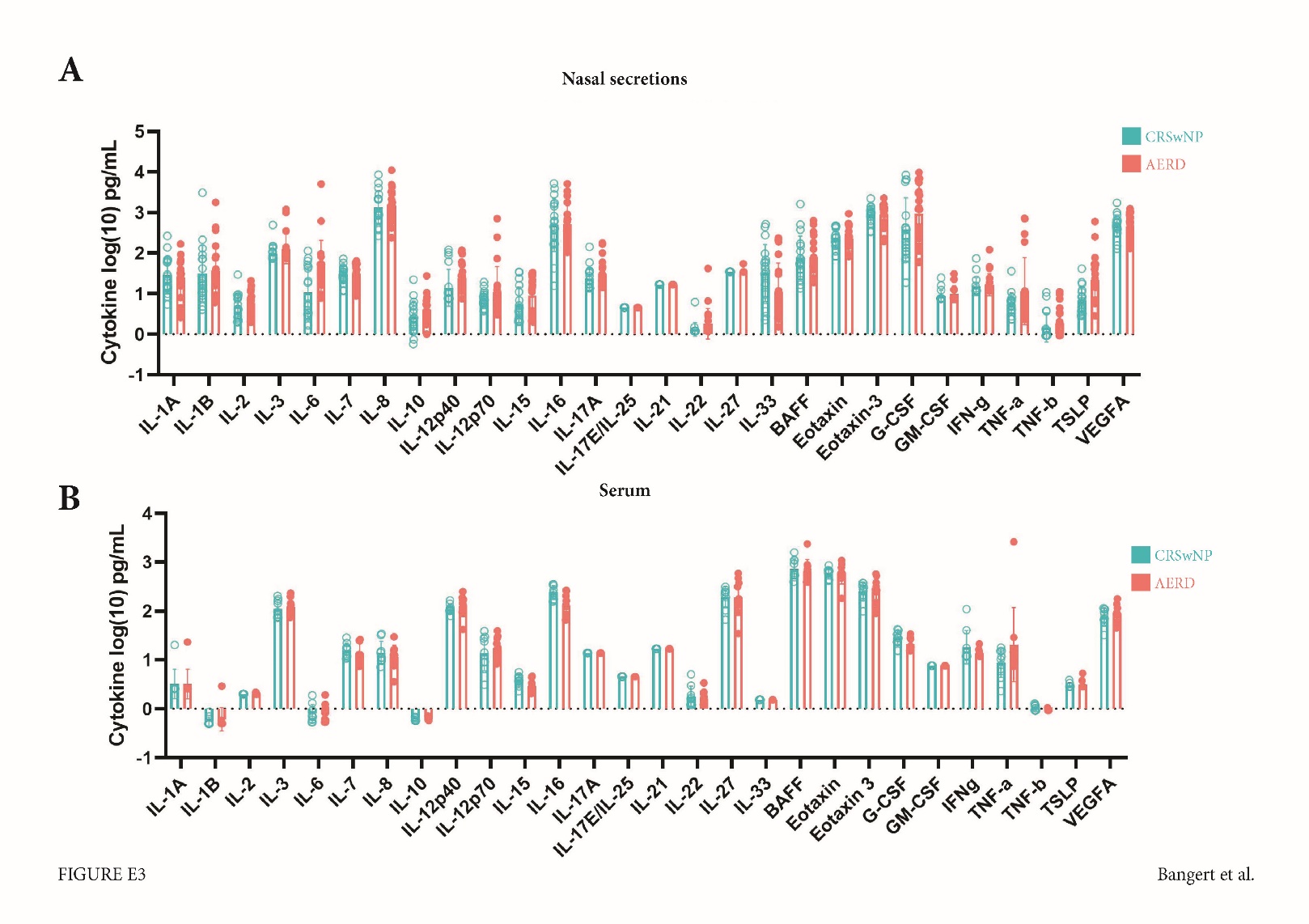
**

**Figure S3: Nasal secretions and serum of patients with AERD and CRSwNP analysed by multiplex mesoscale discovery U-plex detection.** (A, B) IL-1A, IL-1B, IL-2, IL-3, IL-6, IL-7, IL-8, IL-9, IL-10, IL12p40, IL-12p70, IL-15, IL-16, IL-17A, IL-17E/IL-25, IL-21, IL-22, IL-27, IL-33, BAFF, Eotaxin, Eotaxin 3, G-CSF, GM-CSF, IFN-γ, TNF-α, TNF-β, TSLP and VEGFA (y-axes, pg/ml) in (A) nasal secretions or (B) serum of patients suffering from CRSwNP (blue, n=9) or AERD (red, n=10) quantified by mesoscale discovery U-plex detection. Cytokine concentrations were log-transformed for analysis. No statistically significant differences (p-value <0.05) were determined using multiple unpaired t tests with Welch correction per row and Holm-Sidak correction. AERD: aspirin-exacerbated respiratory disease; CRSwNP: chronic rhinosinusitis with nasal polyps.

**Figure S4: scRNA-seq results of individual nasal polyp biopsies per patient**. UMAP plots of all samples as used for analysis. Patient numbers correspond to individuals with AERD and CRSwNP as listed in Table E1. AERD: aspirin-exacerbated respiratory disease; CRSwNP: chronic rhinosinusitis with nasal polyps.

** Figure S5: Gene expression of selected cytokines and chemokines.** Feature plots identifying expression of (A) IL13, (B) IL4, (C) TNF, (D) IL10, (E) CCL17 and (F) CCL22 represented by UMAP in (left) AERD as compared to (right) CRSwNP. Red color displays intensity levels of natural log transformed gene expression. AERD: aspirin-exacerbated respiratory disease; CRSwNP: chronic rhinosinusitis with nasal polyps.

**Figure S6: Differentially expressed genes in T-cell clusters of combined AERD and CRSwNP samples.** Color-coded combined violin plots showing natural log transformed normalized gene expression for (A) TRAC, (B) IFNG, (C) GZMB, (D) LTB, (E) KLF2, (F) KLRG1, (G) IL7R, (H) KLRB1, (I) IL17A, (J) IL26, and (K) FOXP3 in T-cell clusters. AERD: aspirin-exacerbated respiratory disease; CRSwNP: chronic rhinosinusitis with nasal polyps.

**Figure S7: Differentially expressed stress-related genes in AERD and CRSwNP**. (A) Color-coded violin plots showing natural log transformed normalized gene expression of MTRNR2L12 in all clusters in (left) AERD versus (right) CRSwNP. (B, C) Color-coded violin plots showing upregulated normalized gene expression of (B) MTRNR2L8 and (C) MTRNR2L12 in AERD as compared to CRSwNP patients. Each violin graph represents an individual patient. AERD: aspirin-exacerbated respiratory disease; CRSwNP: chronic rhinosinusitis with nasal polyps.
