# Supplementary material for: Comprehensive Analysis of Nasal Polyps Reveals a More Pronounced Type 2 Transcriptomic Profile of Epithelial Cells and Mast Cells in Aspirin-Exacerbated Respiratory Disease
Source: Front Immunol. 2022 Mar 28;13:850494. doi: 10.3389/fimmu.2022.850494 (PMC8996080; doi:10.3389/fimmu.2022.850494)
Supplement: Supplementary file 1 [file DataSheet_1.zip › Supplementary Material/Supplementary Tables 1-6.docx]

**Supplementary tables S1-S6:**


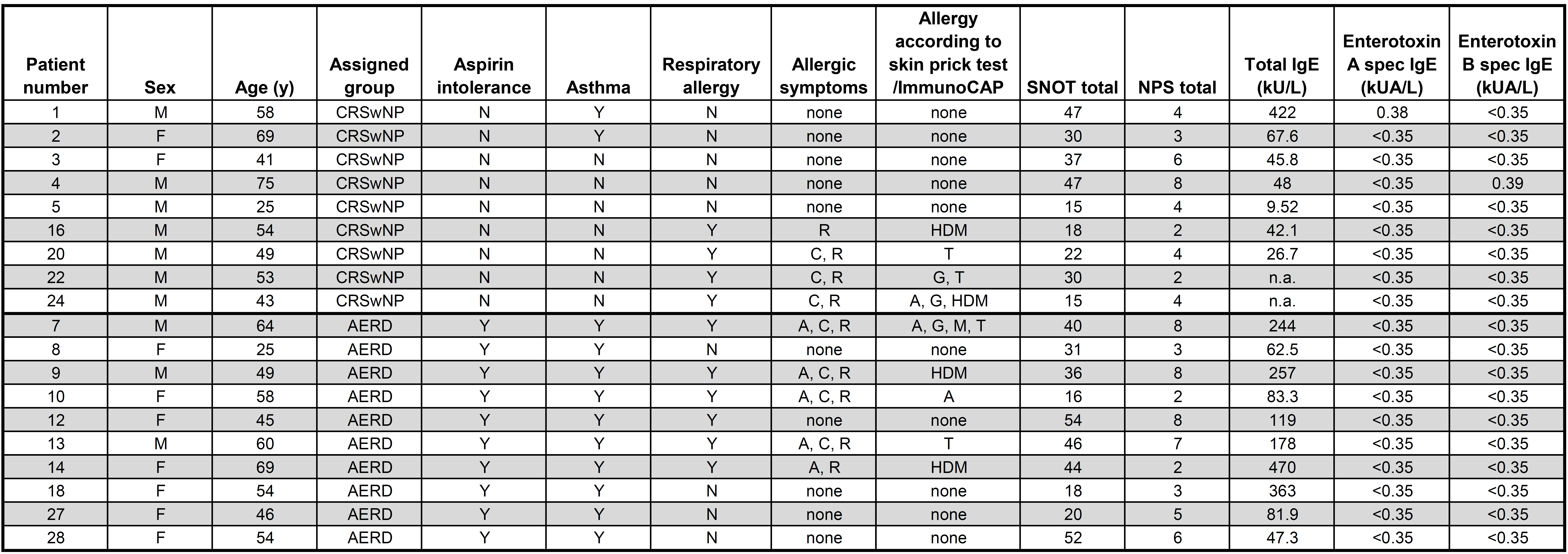


**Table S1 Demographics of individual patients.** Sex: M: Male, F: Female; Assigned group: CRSwNP: Chronic rhinosinusitis with nasal polyps; AERD: Aspirin exacerbated respiratory disease; Aspirin intolerance, Asthma, Respiratory Allergy: Y=Yes, N=No; Allergic symptoms: A=Asthma, C=Conjunctivitis, R=Rhinitis; Allergy: A=Animal dander, G= Grass, HDM= House dust mite; M=Mould, T=Tree; NPS=Nasal polyp score

| **Patient number** | **Assigned group** | **7AAD-CD45-** | **7AAD-CD45+** | | | |
| --- | --- | --- | --- | --- | --- | --- |
|  |  |  | **T** | **Gr** | **Mo/Mac** | **other Lyc** |
| 1 | CRSwNP | 9000 | 3000 | 2000 | 3000 | 1754 |
| 2 | CRSwNP | 9000 | 3000 | 2000 | 3000 | 2928 |
| 3 | CRSwNP | 9000 | 3000 | 1800 | 2900 | 1800 |
| 4 | CRSwNP | 6900 | 2120 | 1325 | 2120 | 1325 |
| 5 | CRSwNP | 9000 | 2880 | 1800 | 2880 | 1800 |
| 7 | AERD | 9000 | 2880 | 1800 | 2880 | 1800 |
| 8 | AERD | 9000 | 2880 | 1800 | 2880 | 1800 |
| 9 | AERD | 9000 | 2880 | 1800 | 2880 | 1800 |
| 10 | AERD | 9000 | 2880 | 1800 | 2880 | 1800 |
| 12 | AERD | 9000 | 2900 | 1800 | 2880 | 1800 |
| 13 | AERD | 9000 | 2980 | 1800 | 2880 | 1800 |
| 14 | AERD | 9000 | 2900 | 1800 | 2880 | 1800 |
| 16 | CRSwNP | 9000 | 2880 | 1800 | 2880 | 1800 |
| 18 | AERD | 9000 | 2880 | 1800 | 2880 | 1800 |
| 20 | CRSwNP | 9000 | 2880 | 1800 | 2880 | 1800 |
| 22 | CRSwNP | 9000 | 2880 | 1800 | 2880 | 1800 |
| 24 | CRSwNP | 9000 | 2880 | 1800 | 2880 | 1800 |
| 27 | AERD | 9000 | 2880 | 1800 | 2880 | 1800 |
| 28 | AERD | 9000 | 2880 | 1800 | 2880 | 1800 |

**Table S2: Cell numbers as sorted by flow cytometry from each biopsy for scRNA-sequencing.** CRSwNP: Chronic rhinosinusitis with nasal polyps; AERD: Aspirin-exacerbated respiratory disease; T: T cells; Gr: Granulocytes; Mo/Mac: Monocytes/Macrophages; other Lyc: other Lymphocytes

| **Patient Number** | **Assigned group** | **Number of cells** | **Median number of reads** | **Median number of expressed genes** |
| --- | --- | --- | --- | --- |
| 1 | CRSwNP | 1125 | 3049 | 1089 |
| 2 | CRSwNP | 1937 | 3114 | 1067 |
| 3 | CRSwNP | 2966 | 1865.5 | 715 |
| 4 | CRSwNP | 2073 | 3120 | 1064 |
| 5 | CRSwNP | 2405 | 3546 | 1133 |
| 7 | AERD | 1419 | 2230 | 730 |
| 8 | AERD | 1551 | 1329 | 427 |
| 9 | AERD | 1243 | 1494 | 475 |
| 10 | AERD | 2950 | 4755.5 | 1622 |
| 12 | AERD | 1856 | 5118.5 | 1714 |
| 13 | AERD | 1980 | 5109 | 1601 |
| 14 | AERD | 2870 | 4084 | 1484.5 |
| 16 | CRSwNP | 2490 | 4782.5 | 1685 |
| 18 | AERD | 2064 | 4922.55 | 1644 |
| 20 | CRSwNP | 3282 | 4563.5 | 1541 |
| 22 | CRSwNP | 2670 | 4486.5 | 1642 |
| 24 | CRSwNP | 3505 | 3749 | 1442 |
| 27 | AERD | 2904 | 4716.5 | 1587 |
| 28 | AERD | 1578 | 6368.5 | 1932 |

**Table S3: Cell numbers, median read and gene numbers** for each biopsy used for scRNA-sequencing before quality control. CRSwNP: Chronic rhinosinusitis with nasal polyps; AERD: Aspirin exacerbated respiratory disease

| **Patient Number** | **Assigned group** | **TC1** | **TC2** | **NK1** | **MyC1** | **MC1** | **MC2** | **NK2** | **TC3** | **TC4** | **MC3** | **TC5** | **TC6** | **TREG** | **BC** | **MyC2** | **pDC** | **EpiC** | **ILC** | **proLy** |
| --- | --- | --- | --- | --- | --- | --- | --- | --- | --- | --- | --- | --- | --- | --- | --- | --- | --- | --- | --- | --- |
| 1 | CRSwNP | 133 | 43 | 94 | 58 | 85 | 34 | 99 | 29 | 65 | 40 | 36 | 16 | 22 | 10 | 16 | 13 | 4 | 39 | 4 |
| 2 | CRSwNP | 173 | 135 | 146 | 191 | 227 | 207 | 80 | 56 | 85 | 133 | 48 | 50 | 44 | 26 | 39 | 6 | 9 | 5 | 7 |
| 3 | CRSwNP | 341 | 343 | 338 | 191 | 241 | 309 | 84 | 86 | 73 | 139 | 77 | 104 | 74 | 108 | 84 | 6 | 70 | 16 | 10 |
| 4 | CRSwNP | 130 | 110 | 186 | 88 | 66 | 53 | 57 | 67 | 75 | 27 | 94 | 32 | 41 | 30 | 15 | 15 | 33 | 14 | 12 |
| 5 | CRSwNP | 226 | 158 | 123 | 342 | 96 | 24 | 147 | 95 | 192 | 17 | 73 | 76 | 57 | 132 | 93 | 140 | 8 | 6 | 22 |
| 7 | AERD | 102 | 175 | 184 | 31 | 38 | 7 | 99 | 137 | 52 | 13 | 85 | 42 | 63 | 37 | 7 | 4 | 20 | 18 | 11 |
| 8 | AERD | 171 | 255 | 82 | 51 | 71 | 26 | 48 | 68 | 59 | 25 | 78 | 120 | 66 | 130 | 16 | 16 | 6 | 18 | 1 |
| 9 | AERD | 121 | 146 | 114 | 8 | 45 | 23 | 31 | 66 | 42 | 18 | 160 | 46 | 37 | 31 | 8 | 6 | 13 | 6 | 11 |
| 10 | AERD | 448 | 154 | 197 | 113 | 175 | 251 | 152 | 96 | 133 | 161 | 91 | 43 | 71 | 71 | 44 | 8 | 8 | 43 | 21 |
| 12 | AERD | 225 | 202 | 183 | 35 | 44 | 12 | 86 | 86 | 79 | 19 | 25 | 46 | 55 | 23 | 8 | 14 | 13 | 15 | 20 |
| 13 | AERD | 233 | 274 | 230 | 25 | 45 | 18 | 78 | 71 | 50 | 23 | 129 | 152 | 86 | 61 | 4 | 5 | 2 | 1 | 11 |
| 14 | AERD | 401 | 121 | 130 | 66 | 204 | 195 | 162 | 78 | 110 | 131 | 40 | 32 | 87 | 6 | 33 | 16 | 78 | 13 | 6 |
| 16 | CRSwNP | 389 | 239 | 126 | 234 | 45 | 12 | 146 | 119 | 128 | 30 | 49 | 65 | 75 | 34 | 36 | 39 | 47 | 33 | 19 |
| 18 | AERD | 177 | 143 | 193 | 53 | 23 | 9 | 66 | 106 | 125 | 17 | 21 | 44 | 53 | 59 | 25 | 24 | 41 | 19 | 24 |
| 20 | CRSwNP | 322 | 166 | 168 | 131 | 410 | 454 | 105 | 103 | 171 | 316 | 88 | 60 | 51 | 54 | 51 | 2 | 15 | 11 | 12 |
| 22 | CRSwNP | 414 | 153 | 238 | 117 | 219 | 154 | 163 | 92 | 60 | 139 | 106 | 44 | 42 | 44 | 40 | 11 | 32 | 18 | 12 |
| 24 | CRSwNP | 546 | 162 | 175 | 506 | 51 | 9 | 332 | 160 | 121 | 19 | 59 | 39 | 89 | 83 | 210 | 107 | 26 | 17 | 33 |
| 27 | AERD | 194 | 168 | 149 | 66 | 192 | 265 | 45 | 197 | 63 | 173 | 142 | 59 | 62 | 52 | 37 | 30 | 11 | 14 | 17 |
| 28 | AERD | 192 | 179 | 122 | 78 | 50 | 40 | 41 | 104 | 74 | 33 | 45 | 78 | 57 | 27 | 33 | 10 | 3 | 7 | 16 |

**Table S4: Absolute cell numbers per patient and cell clusters after quality control filtering, as used for figures and all calculations based on scRNA-seq.** CRSwNP: chronic rhinosinusitis with nasal polyps; AERD: aspirin-exacerbated respiratory disease

| **Gene** | **p_val** | **Avg_logFC** | **Pct.1** | **Pct.2** | **p_val_adj** | **Cluster#** | **Cluster Name** |
| --- | --- | --- | --- | --- | --- | --- | --- |
| CD8A | 0 | 1,62370963 | 0,844 | 0,148 | 0 | 0 | TC1 |
| CCL5 | 0 | 1,42861936 | 0,997 | 0,436 | 0 | 0 |  |
| CD8B | 0 | 1,21841394 | 0,685 | 0,088 | 0 | 0 |  |
| CD3D | 0 | 1,20227754 | 0,98 | 0,396 | 0 | 0 |  |
| CD3G | 0 | 1,18255101 | 0,857 | 0,297 | 0 | 0 |  |
| LINC01871 | 0 | 1,08204732 | 0,758 | 0,226 | 0 | 0 |  |
| KLRC1 | 0 | 1,06227304 | 0,435 | 0,102 | 0 | 0 |  |
| CD2 | 0 | 1,04765344 | 0,905 | 0,401 | 0 | 0 |  |
| ITGA1 | 0 | 1,00318924 | 0,504 | 0,053 | 0 | 0 |  |
| TRGC2 | 0 | 0,96306047 | 0,497 | 0,11 | 0 | 0 |  |
| CLEC2D | 0 | 0,93649818 | 0,731 | 0,295 | 0 | 0 |  |
| TRAC | 0 | 0,86708511 | 0,873 | 0,385 | 0 | 0 |  |
| TRBC2 | 0 | 0,85987851 | 0,842 | 0,473 | 0 | 0 |  |
| CD7 | 0 | 0,85494281 | 0,903 | 0,441 | 0 | 0 |  |
| LAG3 | 0 | 0,83667622 | 0,455 | 0,083 | 0 | 0 |  |
| CXCR6 | 0 | 0,83300036 | 0,494 | 0,123 | 0 | 0 |  |
| LINC02446 | 0 | 0,82552385 | 0,253 | 0,019 | 0 | 0 |  |
| CD3E | 0 | 0,82012688 | 0,864 | 0,398 | 0 | 0 |  |
| LDLRAD4 | 0 | 0,81429196 | 0,582 | 0,257 | 0 | 0 |  |
| ZFP36L2 | 0 | 0,8106275 | 0,949 | 0,751 | 0 | 0 |  |
| ZNF683 | 0 | 0,80892029 | 0,341 | 0,025 | 0 | 0 |  |
| SPRY1 | 0 | 0,78713538 | 0,353 | 0,09 | 0 | 0 |  |
| PTPN22 | 0 | 0,78253877 | 0,589 | 0,248 | 0 | 0 |  |
| GPR171 | 0 | 0,75355558 | 0,509 | 0,159 | 0 | 0 |  |
| KLRC2 | 0 | 0,71182506 | 0,273 | 0,044 | 0 | 0 |  |
| CD96 | 0 | 0,71180057 | 0,603 | 0,24 | 0 | 0 |  |
| GZMA | 0 | 0,69131268 | 0,875 | 0,411 | 0 | 0 |  |
| LEPROTL1 | 0 | 0,68275986 | 0,848 | 0,573 | 0 | 0 |  |
| RUNX3 | 0 | 0,6715932 | 0,686 | 0,4 | 0 | 0 |  |
| GABARAPL1 | 0 | 0,65414708 | 0,6 | 0,331 | 0 | 0 |  |
| LTB | 0 | 1,379349 | 0,854 | 0,305 | 0 | 1 | TC2 |
| IL7R | 0 | 1,27187177 | 0,811 | 0,281 | 0 | 1 |  |
| KLF2 | 3,084E-237 | 0,8678743 | 0,537 | 0,3 | 1,034E-232 | 1 |  |
| CCR7 | 0 | 0,78684206 | 0,382 | 0,087 | 0 | 1 |  |
| ITGB1 | 2,307E-189 | 0,60701641 | 0,559 | 0,357 | 7,737E-185 | 1 |  |
| LDHB | 0 | 0,59737545 | 0,808 | 0,605 | 0 | 1 |  |
| NOSIP | 4,944E-242 | 0,59638674 | 0,494 | 0,255 | 1,658E-237 | 1 |  |
| RPS18 | 0 | 0,59153558 | 0,999 | 0,998 | 0 | 1 |  |
| RGCC | 3,634E-258 | 0,5825669 | 0,74 | 0,444 | 1,219E-253 | 1 |  |
| RPL36A | 6,789E-209 | 0,57913571 | 0,911 | 0,772 | 2,277E-204 | 1 |  |
| RPS12 | 0 | 0,57358517 | 0,999 | 0,996 | 0 | 1 |  |
| RPL36 | 0 | 0,57036418 | 0,999 | 0,988 | 0 | 1 |  |
| RPL31 | 1,111E-169 | 0,56583711 | 0,986 | 0,951 | 3,725E-165 | 1 |  |
| CRIP1 | 8,598E-175 | 0,56509517 | 0,624 | 0,388 | 2,884E-170 | 1 |  |
| RPL34 | 0 | 0,56370174 | 0,999 | 0,999 | 0 | 1 |  |
| RPL13 | 0 | 0,55401372 | 1 | 0,999 | 0 | 1 |  |
| RPL18A | 0 | 0,54833614 | 0,999 | 0,996 | 0 | 1 |  |
| RPL13A | 0 | 0,5467456 | 0,999 | 0,996 | 0 | 1 |  |
| RPS27 | 0 | 0,54161413 | 1 | 0,999 | 0 | 1 |  |
| RPL32 | 0 | 0,53510613 | 0,999 | 0,998 | 0 | 1 |  |
| RPS29 | 0 | 0,53483193 | 1 | 0,998 | 0 | 1 |  |
| TOB1 | 1,311E-225 | 0,53438823 | 0,506 | 0,257 | 4,395E-221 | 1 |  |
| RPS25 | 0 | 0,53224297 | 0,998 | 0,992 | 0 | 1 |  |
| RPS21 | 0 | 0,5257694 | 0,995 | 0,972 | 0 | 1 |  |
| RPLP2 | 0 | 0,51252117 | 0,999 | 0,997 | 0 | 1 |  |
| RPS15A | 0 | 0,51219083 | 0,999 | 0,996 | 0 | 1 |  |
| RPS14 | 0 | 0,51041442 | 0,999 | 0,998 | 0 | 1 |  |
| RPS16 | 0 | 0,5022376 | 0,998 | 0,981 | 0 | 1 |  |
| RPS10 | 6,127E-296 | 0,50162898 | 0,965 | 0,886 | 2,055E-291 | 1 |  |
| RPL39 | 0 | 0,49916107 | 1 | 0,998 | 0 | 1 |  |
| GNLY | 0 | 2,1971477 | 0,945 | 0,2 | 0 | 2 | NK1 |
| FGFBP2 | 0 | 2,11717178 | 0,778 | 0,044 | 0 | 2 |  |
| PRF1 | 0 | 2,00077492 | 0,923 | 0,284 | 0 | 2 |  |
| FCGR3A | 0 | 1,9684693 | 0,82 | 0,081 | 0 | 2 |  |
| NKG7 | 0 | 1,9411835 | 0,999 | 0,461 | 0 | 2 |  |
| SPON2 | 0 | 1,88945414 | 0,751 | 0,093 | 0 | 2 |  |
| GZMB | 0 | 1,67335169 | 0,985 | 0,352 | 0 | 2 |  |
| KLRF1 | 0 | 1,63336872 | 0,8 | 0,069 | 0 | 2 |  |
| CST7 | 0 | 1,53844289 | 0,975 | 0,447 | 0 | 2 |  |
| CTSW | 0 | 1,49998944 | 0,941 | 0,416 | 0 | 2 |  |
| CD247 | 0 | 1,47663679 | 0,845 | 0,326 | 0 | 2 |  |
| KLRD1 | 0 | 1,43788373 | 0,908 | 0,247 | 0 | 2 |  |
| PLAC8 | 0 | 1,41379141 | 0,838 | 0,203 | 0 | 2 |  |
| CCL4 | 0 | 1,32359918 | 0,969 | 0,536 | 0 | 2 |  |
| CLIC3 | 0 | 1,2691641 | 0,665 | 0,139 | 0 | 2 |  |
| AKR1C3 | 0 | 1,26087602 | 0,502 | 0,022 | 0 | 2 |  |
| HOPX | 0 | 1,24477506 | 0,803 | 0,283 | 0 | 2 |  |
| ITGB2 | 0 | 1,23260165 | 0,837 | 0,426 | 0 | 2 |  |
| CCL3 | 0 | 1,22710031 | 0,552 | 0,179 | 0 | 2 |  |
| TRDC | 0 | 1,21101417 | 0,676 | 0,098 | 0 | 2 |  |
| IGFBP7 | 0 | 1,17447815 | 0,52 | 0,054 | 0 | 2 |  |
| GZMM | 0 | 1,08237362 | 0,852 | 0,347 | 0 | 2 |  |
| S1PR5 | 0 | 1,04515723 | 0,48 | 0,03 | 0 | 2 |  |
| EFHD2 | 0 | 1,02847117 | 0,742 | 0,379 | 0 | 2 |  |
| MYOM2 | 0 | 1,02224964 | 0,224 | 0,021 | 0 | 2 |  |
| IFITM2 | 0 | 0,98596843 | 0,96 | 0,743 | 0 | 2 |  |
| ABHD17A | 0 | 0,97080884 | 0,672 | 0,336 | 0 | 2 |  |
| PFN1 | 0 | 0,96264808 | 0,998 | 0,962 | 0 | 2 |  |
| XCL2 | 0 | 0,95788876 | 0,491 | 0,13 | 0 | 2 |  |
| NCR3 | 0 | 0,95784384 | 0,526 | 0,111 | 0 | 2 |  |
| S100A8 | 0 | 4,36684122 | 0,932 | 0,029 | 0 | 3 | MyC1 |
| S100A9 | 0 | 4,2377499 | 0,962 | 0,035 | 0 | 3 |  |
| IL1B | 0 | 4,04944108 | 0,912 | 0,051 | 0 | 3 |  |
| LYZ | 0 | 3,6126391 | 0,961 | 0,055 | 0 | 3 |  |
| G0S2 | 0 | 3,53173736 | 0,841 | 0,039 | 0 | 3 |  |
| CXCL8 | 0 | 3,28368007 | 0,848 | 0,073 | 0 | 3 |  |
| CXCL2 | 0 | 3,14377418 | 0,789 | 0,049 | 0 | 3 |  |
| EREG | 0 | 3,13459898 | 0,853 | 0,028 | 0 | 3 |  |
| SOD2 | 0 | 2,89208122 | 0,938 | 0,143 | 0 | 3 |  |
| CCL20 | 0 | 2,86410096 | 0,525 | 0,053 | 0 | 3 |  |
| PLAUR | 0 | 2,85746262 | 0,967 | 0,194 | 0 | 3 |  |
| CXCL3 | 0 | 2,72496984 | 0,568 | 0,036 | 0 | 3 |  |
| FCN1 | 0 | 2,6811671 | 0,911 | 0,01 | 0 | 3 |  |
| AIF1 | 0 | 2,58823937 | 0,966 | 0,085 | 0 | 3 |  |
| VCAN | 0 | 2,48841896 | 0,846 | 0,009 | 0 | 3 |  |
| IER3 | 0 | 2,46787766 | 0,897 | 0,159 | 0 | 3 |  |
| TIMP1 | 0 | 2,46753373 | 0,957 | 0,397 | 0 | 3 |  |
| CCL3L1 | 0 | 2,40280173 | 0,492 | 0,064 | 0 | 3 |  |
| THBS1 | 0 | 2,3781126 | 0,697 | 0,049 | 0 | 3 |  |
| NAMPT | 0 | 2,31969578 | 0,977 | 0,341 | 0 | 3 |  |
| CD14 | 0 | 2,30838263 | 0,842 | 0,02 | 0 | 3 |  |
| S100A12 | 0 | 2,27306292 | 0,691 | 0,003 | 0 | 3 |  |
| BCL2A1 | 0 | 2,19768532 | 0,938 | 0,232 | 0 | 3 |  |
| C5AR1 | 0 | 2,17212062 | 0,855 | 0,016 | 0 | 3 |  |
| IL1RN | 0 | 2,11408786 | 0,629 | 0,016 | 0 | 3 |  |
| C15orf48 | 0 | 2,10115879 | 0,565 | 0,033 | 0 | 3 |  |
| LST1 | 0 | 2,09648217 | 0,948 | 0,105 | 0 | 3 |  |
| CTSS | 0 | 2,09574721 | 0,952 | 0,367 | 0 | 3 |  |
| OLR1 | 0 | 2,06137822 | 0,734 | 0,02 | 0 | 3 |  |
| MNDA | 0 | 1,99931901 | 0,823 | 0,025 | 0 | 3 |  |
| TPSB2 | 0 | 2,37373878 | 0,997 | 0,205 | 0 | 4 | MC1 |
| TPSAB1 | 0 | 2,34211329 | 1 | 0,19 | 0 | 4 |  |
| CPA3 | 0 | 2,28347203 | 0,995 | 0,133 | 0 | 4 |  |
| CTSG | 0 | 2,18444284 | 0,518 | 0,042 | 0 | 4 |  |
| HPGD | 0 | 2,17836447 | 0,882 | 0,146 | 0 | 4 |  |
| HPGDS | 0 | 1,91339367 | 0,985 | 0,127 | 0 | 4 |  |
| CLU | 0 | 1,91232585 | 0,975 | 0,148 | 0 | 4 |  |
| CD9 | 0 | 1,80728556 | 0,957 | 0,206 | 0 | 4 |  |
| LTC4S | 0 | 1,79153944 | 0,963 | 0,139 | 0 | 4 |  |
| MS4A2 | 0 | 1,76416295 | 0,929 | 0,112 | 0 | 4 |  |
| GATA2 | 0 | 1,65861602 | 0,907 | 0,115 | 0 | 4 |  |
| GLUL | 0 | 1,65631105 | 0,96 | 0,337 | 0 | 4 |  |
| CCL2 | 0 | 1,5616769 | 0,269 | 0,052 | 0 | 4 |  |
| HDC | 0 | 1,5396101 | 0,838 | 0,099 | 0 | 4 |  |
| LMNA | 0 | 1,52678169 | 0,952 | 0,374 | 0 | 4 |  |
| NSMCE1 | 0 | 1,52148625 | 0,865 | 0,242 | 0 | 4 |  |
| IL5 | 0 | 1,48964484 | 0,243 | 0,031 | 0 | 4 |  |
| VWA5A | 0 | 1,48857351 | 0,899 | 0,117 | 0 | 4 |  |
| SLC18A2 | 0 | 1,42256198 | 0,798 | 0,087 | 0 | 4 |  |
| UTS2 | 0 | 1,39188286 | 0,293 | 0,036 | 0 | 4 |  |
| TMEM176B | 0 | 1,37177945 | 0,746 | 0,118 | 0 | 4 |  |
| CAPG | 0 | 1,36094064 | 0,959 | 0,344 | 0 | 4 |  |
| IL1RL1 | 0 | 1,33578533 | 0,822 | 0,108 | 0 | 4 |  |
| ACSL4 | 0 | 1,32698149 | 0,781 | 0,169 | 0 | 4 |  |
| RGS13 | 0 | 1,32252493 | 0,731 | 0,087 | 0 | 4 |  |
| RHEX | 0 | 1,29030121 | 0,768 | 0,097 | 0 | 4 |  |
| CD63 | 0 | 1,2819487 | 0,995 | 0,682 | 0 | 4 |  |
| CPM | 0 | 1,25230643 | 0,724 | 0,102 | 0 | 4 |  |
| LAPTM4A | 0 | 1,23596317 | 0,94 | 0,444 | 0 | 4 |  |
| TSC22D1 | 0 | 1,20422873 | 0,606 | 0,121 | 0 | 4 |  |
| TPSAB1 | 0 | 1,95772268 | 0,997 | 0,196 | 0 | 5 | MC2 |
| TPSB2 | 0 | 1,90393127 | 0,998 | 0,211 | 0 | 5 |  |
| IL13 | 0 | 1,8989436 | 0,433 | 0,036 | 0 | 5 |  |
| GATA2 | 0 | 1,88664005 | 0,919 | 0,12 | 0 | 5 |  |
| CPA3 | 0 | 1,88110895 | 0,993 | 0,139 | 0 | 5 |  |
| GLUL | 0 | 1,74749777 | 0,973 | 0,34 | 0 | 5 |  |
| IL5 | 0 | 1,73788105 | 0,245 | 0,032 | 0 | 5 |  |
| CLU | 0 | 1,69105297 | 0,962 | 0,155 | 0 | 5 |  |
| HPGDS | 0 | 1,6602135 | 0,974 | 0,134 | 0 | 5 |  |
| MS4A2 | 0 | 1,65121621 | 0,934 | 0,118 | 0 | 5 |  |
| AREG | 0 | 1,5823866 | 0,86 | 0,493 | 0 | 5 |  |
| RGS16 | 0 | 1,56315475 | 0,561 | 0,098 | 0 | 5 |  |
| CD9 | 0 | 1,54705119 | 0,976 | 0,211 | 0 | 5 |  |
| LTC4S | 0 | 1,50314708 | 0,944 | 0,146 | 0 | 5 |  |
| LMNA | 0 | 1,49739833 | 0,961 | 0,377 | 0 | 5 |  |
| CCL2 | 2,065E-235 | 1,49088718 | 0,239 | 0,056 | 6,926E-231 | 5 |  |
| HPGD | 0 | 1,43398593 | 0,78 | 0,159 | 0 | 5 |  |
| IL1RL1 | 0 | 1,42577482 | 0,846 | 0,111 | 0 | 5 |  |
| BIRC3 | 0 | 1,38785918 | 0,939 | 0,492 | 0 | 5 |  |
| HDC | 0 | 1,37954685 | 0,794 | 0,107 | 0 | 5 |  |
| NFKBIZ | 0 | 1,37434304 | 0,933 | 0,331 | 0 | 5 |  |
| VWA5A | 0 | 1,37158811 | 0,886 | 0,124 | 0 | 5 |  |
| CPM | 0 | 1,36271255 | 0,737 | 0,105 | 0 | 5 |  |
| SELENOK | 0 | 1,34447475 | 0,994 | 0,801 | 0 | 5 |  |
| LEO1 | 0 | 1,30303317 | 0,742 | 0,17 | 0 | 5 |  |
| LAPTM4A | 0 | 1,29770971 | 0,959 | 0,446 | 0 | 5 |  |
| NSMCE1 | 0 | 1,24467459 | 0,822 | 0,25 | 0 | 5 |  |
| SLC26A2 | 0 | 1,2312428 | 0,626 | 0,095 | 0 | 5 |  |
| SDCBP | 0 | 1,23118376 | 0,967 | 0,593 | 0 | 5 |  |
| ACSL4 | 0 | 1,22477419 | 0,773 | 0,174 | 0 | 5 |  |
| XCL1 | 0 | 1,75227241 | 0,599 | 0,076 | 0 | 6 | NK2 |
| XCL2 | 0 | 1,55581309 | 0,633 | 0,135 | 0 | 6 |  |
| GZMK | 0 | 1,53585279 | 0,722 | 0,133 | 0 | 6 |  |
| TRDC | 0 | 1,5067804 | 0,77 | 0,113 | 0 | 6 |  |
| GZMA | 0 | 1,36462584 | 0,898 | 0,454 | 0 | 6 |  |
| GNLY | 0 | 1,32285879 | 0,642 | 0,248 | 0 | 6 |  |
| KRT86 | 0 | 1,32238169 | 0,407 | 0,02 | 0 | 6 |  |
| CD7 | 0 | 1,26712986 | 0,952 | 0,482 | 0 | 6 |  |
| IL2RB | 0 | 1,23115415 | 0,711 | 0,194 | 0 | 6 |  |
| B3GNT7 | 0 | 1,21805125 | 0,483 | 0,047 | 0 | 6 |  |
| KLRC1 | 0 | 1,17439784 | 0,605 | 0,123 | 0 | 6 |  |
| KRT81 | 0 | 1,11613448 | 0,332 | 0,009 | 0 | 6 |  |
| KLRD1 | 0 | 1,1127184 | 0,848 | 0,277 | 0 | 6 |  |
| KLRF1 | 0 | 1,062745 | 0,572 | 0,112 | 0 | 6 |  |
| APOBEC3G | 0 | 1,03833014 | 0,726 | 0,324 | 0 | 6 |  |
| CLIC3 | 0 | 1,02050504 | 0,598 | 0,163 | 0 | 6 |  |
| PIK3R1 | 0 | 0,99909762 | 0,754 | 0,43 | 0 | 6 |  |
| CEMIP2 | 5,6E-287 | 0,97524554 | 0,613 | 0,3 | 1,878E-282 | 6 |  |
| CD247 | 0 | 0,96482297 | 0,783 | 0,349 | 0 | 6 |  |
| CD160 | 0 | 0,9625377 | 0,396 | 0,071 | 0 | 6 |  |
| DUSP2 | 0 | 0,94546156 | 0,903 | 0,674 | 0 | 6 |  |
| ITM2C | 1,079E-181 | 0,9425899 | 0,5 | 0,271 | 3,62E-177 | 6 |  |
| CMC1 | 1,552E-196 | 0,92658277 | 0,504 | 0,253 | 5,206E-192 | 6 |  |
| KLRB1 | 0 | 0,92606806 | 0,821 | 0,308 | 0 | 6 |  |
| NKG7 | 0 | 0,90059189 | 0,981 | 0,483 | 0 | 6 |  |
| CLDND1 | 6,94E-279 | 0,89802895 | 0,604 | 0,305 | 2,327E-274 | 6 |  |
| MATK | 0 | 0,84174911 | 0,587 | 0,22 | 0 | 6 |  |
| CCL5 | 0 | 0,83760437 | 0,895 | 0,497 | 0 | 6 |  |
| METRNL | 4,336E-178 | 0,83548193 | 0,662 | 0,452 | 1,454E-173 | 6 |  |
| FAM177A1 | 1,29E-218 | 0,81871666 | 0,719 | 0,503 | 4,326E-214 | 6 |  |
| GZMK | 0 | 2,01723741 | 0,846 | 0,13 | 0 | 7 | TC3 |
| CMC1 | 7,647E-234 | 0,88338747 | 0,551 | 0,252 | 2,565E-229 | 7 |  |
| TNFSF9 | 2,028E-301 | 0,87843336 | 0,345 | 0,087 | 6,8E-297 | 7 |  |
| TUBA4A | 8,11E-267 | 0,87766643 | 0,649 | 0,319 | 2,72E-262 | 7 |  |
| CRTAM | 0 | 0,87322446 | 0,337 | 0,059 | 0 | 7 |  |
| DUSP2 | 4,967E-282 | 0,86927519 | 0,888 | 0,677 | 1,666E-277 | 7 |  |
| SH2D1A | 0 | 0,84811006 | 0,514 | 0,15 | 0 | 7 |  |
| CD8B | 0 | 0,82251879 | 0,5 | 0,16 | 0 | 7 |  |
| CD27 | 0 | 0,82020312 | 0,448 | 0,137 | 0 | 7 |  |
| CST7 | 0 | 0,79532797 | 0,912 | 0,474 | 0 | 7 |  |
| CD8A | 2,284E-281 | 0,77121066 | 0,608 | 0,233 | 7,661E-277 | 7 |  |
| CCL5 | 0 | 0,75956836 | 0,986 | 0,494 | 0 | 7 |  |
| CXCR4 | 7,447E-294 | 0,73123421 | 0,957 | 0,755 | 2,498E-289 | 7 |  |
| GZMH | 2,482E-202 | 0,70546024 | 0,582 | 0,264 | 8,323E-198 | 7 |  |
| IFNG | 3,9839E-95 | 0,68715944 | 0,218 | 0,079 | 1,3361E-90 | 7 |  |
| DUSP4 | 1,604E-117 | 0,66511905 | 0,549 | 0,324 | 5,381E-113 | 7 |  |
| CCL4 | 7,878E-215 | 0,66270686 | 0,896 | 0,56 | 2,642E-210 | 7 |  |
| LYAR | 2,068E-183 | 0,6433222 | 0,545 | 0,272 | 6,935E-179 | 7 |  |
| TIGIT | 6,755E-208 | 0,61617947 | 0,433 | 0,158 | 2,266E-203 | 7 |  |
| DNAJB1 | 9,258E-115 | 0,6147585 | 0,619 | 0,42 | 3,105E-110 | 7 |  |
| IL32 | 1,559E-256 | 0,59667424 | 0,953 | 0,544 | 5,228E-252 | 7 |  |
| CCL4L2 | 1,7743E-74 | 0,59514913 | 0,386 | 0,21 | 5,9506E-70 | 7 |  |
| TAGAP | 3,4831E-95 | 0,58554111 | 0,483 | 0,299 | 1,1682E-90 | 7 |  |
| PPP2R5C | 3,658E-161 | 0,58475588 | 0,719 | 0,499 | 1,227E-156 | 7 |  |
| TRAC | 2,724E-222 | 0,57242979 | 0,821 | 0,438 | 9,134E-218 | 7 |  |
| TRAT1 | 1,253E-182 | 0,56953361 | 0,371 | 0,134 | 4,202E-178 | 7 |  |
| RNF19A | 1,484E-111 | 0,56689351 | 0,551 | 0,347 | 4,977E-107 | 7 |  |
| TRBC2 | 1,796E-161 | 0,55823146 | 0,793 | 0,514 | 6,025E-157 | 7 |  |
| RGCC | 2,208E-107 | 0,55245223 | 0,684 | 0,462 | 7,406E-103 | 7 |  |
| KLRG1 | 2,899E-111 | 0,55113277 | 0,344 | 0,158 | 9,721E-107 | 7 |  |
| CXCR6 | 0 | 1,24731319 | 0,665 | 0,152 | 0 | 8 | TC4 |
| IL7R | 0 | 1,09537429 | 0,749 | 0,312 | 0 | 8 |  |
| KLRB1 | 0 | 0,98991909 | 0,712 | 0,319 | 0 | 8 |  |
| CD3D | 0 | 0,98356833 | 0,965 | 0,458 | 0 | 8 |  |
| LINC01871 | 0 | 0,97918661 | 0,736 | 0,283 | 0 | 8 |  |
| CD2 | 0 | 0,96521937 | 0,911 | 0,453 | 0 | 8 |  |
| SPOCK2 | 0 | 0,93939602 | 0,752 | 0,323 | 0 | 8 |  |
| CD3G | 0 | 0,91653055 | 0,848 | 0,356 | 0 | 8 |  |
| TRAC | 0 | 0,91207509 | 0,89 | 0,434 | 0 | 8 |  |
| RORA | 0 | 0,87151028 | 0,723 | 0,342 | 0 | 8 |  |
| CCR6 | 0 | 0,82538494 | 0,398 | 0,065 | 0 | 8 |  |
| CD40LG | 0 | 0,78170958 | 0,343 | 0,047 | 0 | 8 |  |
| TRBC2 | 4,224E-240 | 0,77457761 | 0,838 | 0,512 | 1,417E-235 | 8 |  |
| CD3E | 0 | 0,73541318 | 0,867 | 0,446 | 0 | 8 |  |
| CD6 | 4,709E-264 | 0,6516685 | 0,456 | 0,151 | 1,579E-259 | 8 |  |
| GPR171 | 3,03E-219 | 0,63510395 | 0,503 | 0,196 | 1,016E-214 | 8 |  |
| JAML | 7,838E-212 | 0,62966818 | 0,513 | 0,21 | 2,629E-207 | 8 |  |
| TMIGD2 | 2,215E-186 | 0,61626893 | 0,3 | 0,091 | 7,429E-182 | 8 |  |
| EVL | 4,618E-183 | 0,59179497 | 0,792 | 0,521 | 1,549E-178 | 8 |  |
| PDCD1 | 2,636E-281 | 0,54307386 | 0,3 | 0,066 | 8,84E-277 | 8 |  |
| AC058791.1 | 1,076E-118 | 0,54286586 | 0,537 | 0,31 | 3,609E-114 | 8 |  |
| ODF2L | 2,518E-141 | 0,54126109 | 0,486 | 0,24 | 8,445E-137 | 8 |  |
| MAF | 2,057E-166 | 0,53968114 | 0,356 | 0,129 | 6,899E-162 | 8 |  |
| LCK | 6,457E-189 | 0,53932621 | 0,746 | 0,411 | 2,166E-184 | 8 |  |
| CD96 | 2,579E-177 | 0,5347541 | 0,586 | 0,279 | 8,65E-173 | 8 |  |
| TRBC1 | 1,2024E-61 | 0,52708885 | 0,545 | 0,364 | 4,0326E-57 | 8 |  |
| AC016831.5 | 3,321E-124 | 0,52555334 | 0,52 | 0,286 | 1,114E-119 | 8 |  |
| ICOS | 6,408E-145 | 0,52362771 | 0,356 | 0,137 | 2,149E-140 | 8 |  |
| TTC39C | 5,949E-133 | 0,51081349 | 0,444 | 0,212 | 1,995E-128 | 8 |  |
| TNFRSF25 | 1,328E-225 | 0,51053102 | 0,319 | 0,089 | 4,455E-221 | 8 |  |
| EGR1 | 0 | 1,78688185 | 0,705 | 0,132 | 0 | 9 | MC3 |
| TUBA1A | 2,532E-270 | 1,68690939 | 0,795 | 0,492 | 8,492E-266 | 9 |  |
| TPSAB1 | 0 | 1,67432024 | 0,914 | 0,216 | 0 | 9 |  |
| TPSB2 | 0 | 1,67281533 | 0,906 | 0,231 | 0 | 9 |  |
| JUN | 0 | 1,63608047 | 0,929 | 0,617 | 0 | 9 |  |
| FOSB | 0 | 1,6298836 | 0,974 | 0,576 | 0 | 9 |  |
| MS4A2 | 0 | 1,51700648 | 0,835 | 0,139 | 0 | 9 |  |
| GATA2 | 0 | 1,49957257 | 0,829 | 0,14 | 0 | 9 |  |
| CPA3 | 0 | 1,49356074 | 0,899 | 0,161 | 0 | 9 |  |
| LMNA | 0 | 1,48396547 | 0,916 | 0,391 | 0 | 9 |  |
| LTC4S | 0 | 1,42961821 | 0,861 | 0,167 | 0 | 9 |  |
| TENT5A | 0 | 1,42917623 | 0,655 | 0,139 | 0 | 9 |  |
| CLU | 0 | 1,37324707 | 0,851 | 0,177 | 0 | 9 |  |
| FOS | 7,311E-226 | 1,36597241 | 0,891 | 0,601 | 2,452E-221 | 9 |  |
| CD69 | 0 | 1,35625748 | 0,938 | 0,696 | 0 | 9 |  |
| AREG | 9,558E-148 | 1,34389324 | 0,726 | 0,507 | 3,206E-143 | 9 |  |
| HDC | 0 | 1,34029674 | 0,723 | 0,125 | 0 | 9 |  |
| NR4A1 | 0 | 1,33416346 | 0,842 | 0,32 | 0 | 9 |  |
| CSF1 | 0 | 1,33263461 | 0,506 | 0,12 | 0 | 9 |  |
| HPGD | 0 | 1,30163757 | 0,722 | 0,174 | 0 | 9 |  |
| PPP1R15A | 0 | 1,29328091 | 0,961 | 0,612 | 0 | 9 |  |
| TSC22D1 | 0 | 1,28983561 | 0,53 | 0,138 | 0 | 9 |  |
| HPGDS | 0 | 1,28671585 | 0,874 | 0,156 | 0 | 9 |  |
| DUSP6 | 0 | 1,28505197 | 0,695 | 0,196 | 0 | 9 |  |
| PTGS2 | 0 | 1,27002456 | 0,623 | 0,173 | 0 | 9 |  |
| RGS1 | 0 | 1,2642717 | 0,941 | 0,629 | 0 | 9 |  |
| NFKBIZ | 0 | 1,26212326 | 0,84 | 0,348 | 0 | 9 |  |
| SGK1 | 0 | 1,25755159 | 0,608 | 0,172 | 0 | 9 |  |
| RHEX | 0 | 1,25602571 | 0,701 | 0,119 | 0 | 9 |  |
| GLUL | 0 | 1,25414076 | 0,891 | 0,357 | 0 | 9 |  |
| GZMH | 0 | 1,56821229 | 0,858 | 0,255 | 0 | 10 | TC5 |
| NKG7 | 0 | 1,17428169 | 0,992 | 0,492 | 0 | 10 |  |
| FGFBP2 | 0 | 1,1267082 | 0,593 | 0,094 | 0 | 10 |  |
| GNLY | 0 | 0,96638596 | 0,817 | 0,247 | 0 | 10 |  |
| TRGC2 | 2,409E-204 | 0,92654299 | 0,448 | 0,156 | 8,078E-200 | 10 |  |
| CST7 | 0 | 0,88107843 | 0,943 | 0,478 | 0 | 10 |  |
| IL32 | 0 | 0,799749 | 0,983 | 0,548 | 0 | 10 |  |
| CD3D | 1,218E-271 | 0,72449438 | 0,933 | 0,464 | 4,084E-267 | 10 |  |
| PRF1 | 3,637E-249 | 0,68122138 | 0,742 | 0,328 | 1,22E-244 | 10 |  |
| GZMM | 1,036E-248 | 0,67937294 | 0,786 | 0,378 | 3,476E-244 | 10 |  |
| KLRG1 | 6,298E-205 | 0,67724269 | 0,445 | 0,156 | 2,112E-200 | 10 |  |
| CD52 | 5,111E-153 | 0,67547699 | 0,975 | 0,841 | 1,714E-148 | 10 |  |
| CMC1 | 1,0703E-85 | 0,66083227 | 0,458 | 0,26 | 3,5897E-81 | 10 |  |
| C12orf75 | 5,034E-211 | 0,64830596 | 0,604 | 0,257 | 1,688E-206 | 10 |  |
| CTSW | 7,817E-195 | 0,64422299 | 0,788 | 0,453 | 2,622E-190 | 10 |  |
| HCST | 6,881E-212 | 0,62939528 | 0,959 | 0,812 | 2,308E-207 | 10 |  |
| LYAR | 1,307E-158 | 0,61281258 | 0,569 | 0,274 | 4,383E-154 | 10 |  |
| TRGC1 | 3,3142E-64 | 0,60486828 | 0,218 | 0,089 | 1,1115E-59 | 10 |  |
| PFN1 | 1,294E-192 | 0,59752556 | 0,996 | 0,964 | 4,34E-188 | 10 |  |
| TRBC1 | 4,387E-128 | 0,59661127 | 0,656 | 0,36 | 1,471E-123 | 10 |  |
| ITGB2 | 1,172E-130 | 0,56368443 | 0,704 | 0,455 | 3,932E-126 | 10 |  |
| KLF3 | 2,011E-107 | 0,55447833 | 0,441 | 0,218 | 6,745E-103 | 10 |  |
| TUBA4A | 9,273E-114 | 0,55409967 | 0,582 | 0,326 | 3,11E-109 | 10 |  |
| ITGB1 | 4,1369E-80 | 0,54515442 | 0,557 | 0,37 | 1,3874E-75 | 10 |  |
| S100A4 | 3,254E-145 | 0,51052054 | 0,985 | 0,906 | 1,091E-140 | 10 |  |
| LINC01871 | 2,1594E-86 | 0,50467715 | 0,548 | 0,296 | 7,2421E-82 | 10 |  |
| EMP3 | 8,351E-113 | 0,50006242 | 0,9 | 0,75 | 2,801E-108 | 10 |  |
| S1PR4 | 1,4641E-84 | 0,48417573 | 0,377 | 0,186 | 4,9104E-80 | 10 |  |
| TRAC | 7,472E-108 | 0,48046856 | 0,751 | 0,446 | 2,506E-103 | 10 |  |
| MYL12A | 7,549E-163 | 0,48036169 | 0,945 | 0,902 | 2,532E-158 | 10 |  |
| KLF2 | 1,155E-224 | 1,27644384 | 0,673 | 0,312 | 3,873E-220 | 11 | TC6 |
| CCR7 | 0 | 1,20194864 | 0,624 | 0,098 | 0 | 11 |  |
| SELL | 0 | 1,1111694 | 0,672 | 0,142 | 0 | 11 |  |
| LTB | 0 | 1,01678013 | 0,922 | 0,341 | 0 | 11 |  |
| LEF1 | 0 | 0,86228814 | 0,468 | 0,031 | 0 | 11 |  |
| MAL | 0 | 0,85016782 | 0,483 | 0,039 | 0 | 11 |  |
| LDHB | 6,743E-265 | 0,84967968 | 0,875 | 0,616 | 2,262E-260 | 11 |  |
| NOSIP | 1,556E-207 | 0,82844311 | 0,628 | 0,266 | 5,219E-203 | 11 |  |
| IL7R | 6,281E-253 | 0,77544206 | 0,79 | 0,319 | 2,106E-248 | 11 |  |
| RPL31 | 2,865E-124 | 0,76899201 | 0,998 | 0,953 | 9,608E-120 | 11 |  |
| RPL32 | 0 | 0,76098232 | 1 | 0,998 | 0 | 11 |  |
| RPS12 | 0 | 0,75063566 | 1 | 0,996 | 0 | 11 |  |
| RPS8 | 0 | 0,74535007 | 1 | 0,991 | 0 | 11 |  |
| RPS13 | 0 | 0,74043024 | 1 | 0,993 | 0 | 11 |  |
| RPL34 | 0 | 0,73320465 | 1 | 0,999 | 0 | 11 |  |
| RPS25 | 0 | 0,71150773 | 1 | 0,992 | 0 | 11 |  |
| RPL36 | 0 | 0,70454667 | 1 | 0,989 | 0 | 11 |  |
| RPS27 | 0 | 0,70159307 | 1 | 0,999 | 0 | 11 |  |
| RPL13 | 0 | 0,69173365 | 1 | 0,999 | 0 | 11 |  |
| RPS6 | 0 | 0,69166483 | 1 | 0,993 | 0 | 11 |  |
| RPL21 | 6,432E-269 | 0,69029847 | 1 | 0,998 | 2,157E-264 | 11 |  |
| TSHZ2 | 0 | 0,69017398 | 0,328 | 0,026 | 0 | 11 |  |
| RPLP2 | 0 | 0,68974303 | 1 | 0,997 | 0 | 11 |  |
| RPS18 | 0 | 0,68655543 | 1 | 0,998 | 0 | 11 |  |
| TCF7 | 6,953E-262 | 0,68536621 | 0,499 | 0,14 | 2,332E-257 | 11 |  |
| RPS15A | 0 | 0,68434235 | 1 | 0,996 | 0 | 11 |  |
| RPS14 | 0 | 0,67913802 | 1 | 0,998 | 0 | 11 |  |
| RPS29 | 0 | 0,67079658 | 1 | 0,998 | 0 | 11 |  |
| RPS3A | 0 | 0,66051083 | 0,998 | 0,985 | 0 | 11 |  |
| RPS28 | 0 | 0,66027991 | 1 | 0,998 | 0 | 11 |  |
| TNFRSF4 | 0 | 1,54873433 | 0,532 | 0,141 | 0 | 12 | Treg |
| BATF | 0 | 1,27093785 | 0,543 | 0,151 | 0 | 12 |  |
| CTLA4 | 0 | 1,25735611 | 0,556 | 0,075 | 0 | 12 |  |
| IL2RA | 0 | 1,14455865 | 0,387 | 0,041 | 0 | 12 |  |
| LTB | 7,345E-272 | 1,13069009 | 0,807 | 0,346 | 2,463E-267 | 12 |  |
| IL32 | 6,862E-274 | 1,10017052 | 0,962 | 0,553 | 2,301E-269 | 12 |  |
| TIGIT | 0 | 1,07984804 | 0,576 | 0,159 | 0 | 12 |  |
| LINC01943 | 0 | 1,05182998 | 0,374 | 0,037 | 0 | 12 |  |
| TNFRSF18 | 9,792E-174 | 1,0343492 | 0,491 | 0,186 | 3,284E-169 | 12 |  |
| CD27 | 0 | 0,94058898 | 0,573 | 0,14 | 0 | 12 |  |
| ARID5B | 1,912E-217 | 0,88838506 | 0,649 | 0,267 | 6,413E-213 | 12 |  |
| CORO1B | 6,894E-204 | 0,88286987 | 0,549 | 0,211 | 2,312E-199 | 12 |  |
| TRAC | 3,672E-247 | 0,88044408 | 0,891 | 0,444 | 1,232E-242 | 12 |  |
| LAIR2 | 8,35E-303 | 0,880178 | 0,275 | 0,038 | 2,8E-298 | 12 |  |
| ICOS | 2,716E-242 | 0,8703442 | 0,482 | 0,137 | 9,108E-238 | 12 |  |
| TRBC1 | 6,64E-102 | 0,85870242 | 0,63 | 0,364 | 2,2269E-97 | 12 |  |
| MAF | 1,051E-244 | 0,85575354 | 0,465 | 0,13 | 3,525E-240 | 12 |  |
| TBC1D4 | 0 | 0,84238681 | 0,398 | 0,045 | 0 | 12 |  |
| FOXP3 | 0 | 0,8344909 | 0,336 | 0,004 | 0 | 12 |  |
| UGP2 | 2,14E-118 | 0,7866899 | 0,575 | 0,315 | 7,177E-114 | 12 |  |
| SPOCK2 | 6,028E-174 | 0,77304811 | 0,693 | 0,334 | 2,022E-169 | 12 |  |
| TRBC2 | 1,766E-137 | 0,74255272 | 0,829 | 0,519 | 5,921E-133 | 12 |  |
| PBXIP1 | 5,461E-173 | 0,7194537 | 0,609 | 0,275 | 1,832E-168 | 12 |  |
| DUSP4 | 6,446E-122 | 0,71494391 | 0,631 | 0,326 | 2,162E-117 | 12 |  |
| RTKN2 | 0 | 0,70127441 | 0,209 | 0,009 | 0 | 12 |  |
| ICA1 | 0 | 0,69176945 | 0,239 | 0,011 | 0 | 12 |  |
| DNPH1 | 1,06E-168 | 0,68677827 | 0,419 | 0,138 | 3,556E-164 | 12 |  |
| ZC2HC1A | 0 | 0,67524841 | 0,244 | 0,024 | 0 | 12 |  |
| GATA3 | 2,4497E-97 | 0,65562185 | 0,353 | 0,137 | 8,2157E-93 | 12 |  |
| CD28 | 0 | 0,63717046 | 0,408 | 0,077 | 0 | 12 |  |
| IGKC | 0 | 3,96487835 | 0,761 | 0,311 | 0 | 13 | BC |
| IGHA1 | 2,162E-228 | 3,81362559 | 0,491 | 0,15 | 7,252E-224 | 13 |  |
| IGLC2 | 1,382E-288 | 3,49119712 | 0,402 | 0,087 | 4,634E-284 | 13 |  |
| IGLC3 | 4,178E-237 | 2,74737881 | 0,303 | 0,057 | 1,401E-232 | 13 |  |
| CD79A | 0 | 2,61660179 | 0,902 | 0,013 | 0 | 13 |  |
| IGHM | 0 | 2,61598036 | 0,499 | 0,014 | 0 | 13 |  |
| JCHAIN | 0 | 2,42432527 | 0,389 | 0,054 | 0 | 13 |  |
| IGHG1 | 0 | 2,40348363 | 0,219 | 0,016 | 0 | 13 |  |
| MS4A1 | 0 | 2,31003354 | 0,781 | 0,007 | 0 | 13 |  |
| IGHG3 | 3,327E-302 | 2,12406769 | 0,208 | 0,02 | 1,116E-297 | 13 |  |
| CD74 | 0 | 2,07720948 | 0,985 | 0,565 | 0 | 13 |  |
| HLA-DRA | 0 | 1,77458867 | 0,909 | 0,263 | 0 | 13 |  |
| VPREB3 | 0 | 1,64201521 | 0,528 | 0,002 | 0 | 13 |  |
| HLA-DQA1 | 0 | 1,56336378 | 0,78 | 0,158 | 0 | 13 |  |
| HLA-DQB1 | 0 | 1,54062245 | 0,825 | 0,23 | 0 | 13 |  |
| MEF2C | 0 | 1,48182553 | 0,64 | 0,065 | 0 | 13 |  |
| BANK1 | 0 | 1,47279776 | 0,583 | 0,007 | 0 | 13 |  |
| HLA-DPB1 | 0 | 1,44720331 | 0,892 | 0,386 | 0 | 13 |  |
| HLA-DPA1 | 0 | 1,43282363 | 0,886 | 0,409 | 0 | 13 |  |
| IGHG4 | 1,1576E-32 | 1,4294498 | 0,137 | 0,052 | 3,8825E-28 | 13 |  |
| IGHA2 | 0 | 1,40974324 | 0,218 | 0,005 | 0 | 13 |  |
| TNFRSF13C | 0 | 1,39894691 | 0,576 | 0,011 | 0 | 13 |  |
| HLA-DRB1 | 0 | 1,3339243 | 0,892 | 0,384 | 0 | 13 |  |
| CD79B | 0 | 1,29886617 | 0,542 | 0,055 | 0 | 13 |  |
| LINC01781 | 0 | 1,24572505 | 0,342 | 0 | 0 | 13 |  |
| RALGPS2 | 0 | 1,16459664 | 0,478 | 0,019 | 0 | 13 |  |
| IGHD | 0 | 1,12363513 | 0,25 | 0,004 | 0 | 13 |  |
| HLA-DRB5 | 2,998E-277 | 1,08862117 | 0,474 | 0,118 | 1,005E-272 | 13 |  |
| CD83 | 3,358E-181 | 1,06085151 | 0,66 | 0,286 | 1,126E-176 | 13 |  |
| HLA-DMA | 0 | 1,05211523 | 0,686 | 0,198 | 0 | 13 |  |
| HLA-DRA | 0 | 2,80241703 | 0,994 | 0,266 | 0 | 14 | MyC2 |
| HLA-DQA1 | 0 | 2,76551465 | 0,914 | 0,159 | 0 | 14 |  |
| HLA-DPB1 | 0 | 2,68942955 | 0,977 | 0,387 | 0 | 14 |  |
| HLA-DPA1 | 0 | 2,63049534 | 0,97 | 0,41 | 0 | 14 |  |
| HLA-DRB1 | 0 | 2,59136497 | 0,981 | 0,385 | 0 | 14 |  |
| C1QA | 0 | 2,47427249 | 0,543 | 0,008 | 0 | 14 |  |
| HLA-DQB1 | 0 | 2,42294562 | 0,932 | 0,231 | 0 | 14 |  |
| C1QB | 0 | 2,36355622 | 0,498 | 0,006 | 0 | 14 |  |
| CST3 | 0 | 2,34693571 | 0,98 | 0,28 | 0 | 14 |  |
| CD74 | 0 | 2,31175318 | 0,994 | 0,568 | 0 | 14 |  |
| C1QC | 0 | 2,25496667 | 0,499 | 0,005 | 0 | 14 |  |
| RNASE1 | 0 | 2,13386569 | 0,322 | 0,01 | 0 | 14 |  |
| SPP1 | 0 | 2,06741557 | 0,163 | 0,009 | 0 | 14 |  |
| CTSB | 0 | 2,02503751 | 0,871 | 0,253 | 0 | 14 |  |
| HLA-DRB5 | 0 | 1,91914183 | 0,598 | 0,118 | 0 | 14 |  |
| CTSZ | 0 | 1,90579101 | 0,887 | 0,181 | 0 | 14 |  |
| APOE | 4,895E-182 | 1,87803198 | 0,289 | 0,053 | 1,642E-177 | 14 |  |
| CXCL8 | 0 | 1,87203366 | 0,691 | 0,116 | 0 | 14 |  |
| FTL | 2,297E-291 | 1,83083766 | 1 | 0,972 | 7,705E-287 | 14 |  |
| NPC2 | 0 | 1,76178961 | 0,959 | 0,342 | 0 | 14 |  |
| LYZ | 0 | 1,7585294 | 0,942 | 0,101 | 0 | 14 |  |
| APOC1 | 1,833E-196 | 1,74846841 | 0,38 | 0,082 | 6,147E-192 | 14 |  |
| MS4A6A | 0 | 1,73669119 | 0,81 | 0,076 | 0 | 14 |  |
| C15orf48 | 0 | 1,73615493 | 0,626 | 0,058 | 0 | 14 |  |
| AIF1 | 0 | 1,69114091 | 0,947 | 0,13 | 0 | 14 |  |
| HLA-DMA | 0 | 1,63735241 | 0,897 | 0,196 | 0 | 14 |  |
| IER3 | 0 | 1,6227835 | 0,748 | 0,2 | 0 | 14 |  |
| INSIG1 | 0 | 1,55815271 | 0,792 | 0,251 | 0 | 14 |  |
| CTSL | 0 | 1,54563533 | 0,434 | 0,065 | 0 | 14 |  |
| SDS | 0 | 1,50609274 | 0,419 | 0,011 | 0 | 14 |  |
| PTGDS | 0 | 2,71921299 | 0,4 | 0,025 | 0 | 15 | PDC |
| IRF7 | 0 | 2,67454417 | 0,975 | 0,158 | 0 | 15 |  |
| JCHAIN | 0 | 2,67410586 | 0,975 | 0,051 | 0 | 15 |  |
| SOX4 | 0 | 2,4145389 | 0,854 | 0,087 | 0 | 15 |  |
| PLD4 | 0 | 2,33172528 | 0,966 | 0,019 | 0 | 15 |  |
| PPP1R14B | 0 | 2,32640335 | 0,958 | 0,213 | 0 | 15 |  |
| IRF8 | 0 | 2,30632559 | 0,953 | 0,104 | 0 | 15 |  |
| TCF4 | 0 | 2,21931609 | 0,953 | 0,044 | 0 | 15 |  |
| GPR183 | 1,146E-302 | 2,18330352 | 0,983 | 0,463 | 3,843E-298 | 15 |  |
| CD74 | 1,831E-272 | 2,04095791 | 0,998 | 0,572 | 6,14E-268 | 15 |  |
| LILRA4 | 0 | 2,02902327 | 0,917 | 0,003 | 0 | 15 |  |
| CCDC50 | 0 | 2,00480505 | 0,932 | 0,076 | 0 | 15 |  |
| SERPINF1 | 0 | 1,96722417 | 0,939 | 0,038 | 0 | 15 |  |
| ITM2C | 0 | 1,95281401 | 0,964 | 0,276 | 0 | 15 |  |
| IL3RA | 0 | 1,9515577 | 0,922 | 0,03 | 0 | 15 |  |
| APP | 0 | 1,92922109 | 0,93 | 0,057 | 0 | 15 |  |
| MZB1 | 0 | 1,90616003 | 0,922 | 0,031 | 0 | 15 |  |
| TSPAN13 | 0 | 1,84170819 | 0,888 | 0,021 | 0 | 15 |  |
| PLAC8 | 0 | 1,80221297 | 0,972 | 0,255 | 0 | 15 |  |
| NPC2 | 0 | 1,78924951 | 0,979 | 0,348 | 0 | 15 |  |
| GZMB | 5,872E-276 | 1,77353827 | 0,983 | 0,406 | 1,969E-271 | 15 |  |
| RASD1 | 0 | 1,74837542 | 0,576 | 0,013 | 0 | 15 |  |
| IRF4 | 0 | 1,74669772 | 0,871 | 0,072 | 0 | 15 |  |
| DERL3 | 0 | 1,74213306 | 0,89 | 0,011 | 0 | 15 |  |
| C12orf75 | 0 | 1,7039297 | 0,972 | 0,262 | 0 | 15 |  |
| PTCRA | 0 | 1,64103641 | 0,852 | 0,001 | 0 | 15 |  |
| TCL1A | 0 | 1,63774592 | 0,339 | 0,003 | 0 | 15 |  |
| UGCG | 0 | 1,6058497 | 0,883 | 0,09 | 0 | 15 |  |
| SCT | 0 | 1,5969241 | 0,788 | 0,001 | 0 | 15 |  |
| SEC61B | 8,896E-291 | 1,58717655 | 0,994 | 0,697 | 2,984E-286 | 15 |  |
| SLPI | 0 | 3,78682111 | 0,854 | 0,039 | 0 | 16 | EpiC |
| S100A2 | 0 | 3,63143401 | 0,686 | 0,02 | 0 | 16 |  |
| KRT17 | 0 | 3,56293546 | 0,681 | 0,015 | 0 | 16 |  |
| SERPINB3 | 0 | 3,41192107 | 0,651 | 0,022 | 0 | 16 |  |
| WFDC2 | 0 | 3,39437496 | 0,784 | 0,021 | 0 | 16 |  |
| TFF3 | 0 | 3,27446098 | 0,499 | 0,028 | 0 | 16 |  |
| KRT19 | 0 | 3,1715577 | 0,831 | 0,056 | 0 | 16 |  |
| TACSTD2 | 0 | 2,94079148 | 0,822 | 0,015 | 0 | 16 |  |
| MMP10 | 0 | 2,59789364 | 0,431 | 0,005 | 0 | 16 |  |
| KRT18 | 0 | 2,5635388 | 0,806 | 0,014 | 0 | 16 |  |
| POSTN | 0 | 2,5381784 | 0,551 | 0,003 | 0 | 16 |  |
| BPIFB1 | 1,38E-217 | 2,51446623 | 0,21 | 0,015 | 4,63E-213 | 16 |  |
| CST1 | 5,726E-259 | 2,37895156 | 0,205 | 0,011 | 1,92E-254 | 16 |  |
| AQP3 | 6,461E-281 | 2,37772141 | 0,777 | 0,198 | 2,167E-276 | 16 |  |
| KRT8 | 0 | 2,35070193 | 0,784 | 0,009 | 0 | 16 |  |
| SFN | 0 | 2,32253859 | 0,658 | 0,005 | 0 | 16 |  |
| ELF3 | 0 | 2,32160689 | 0,711 | 0,007 | 0 | 16 |  |
| CXCL1 | 0 | 2,31605594 | 0,385 | 0,02 | 0 | 16 |  |
| MT1X | 3,227E-224 | 2,2960456 | 0,772 | 0,231 | 1,082E-219 | 16 |  |
| VMO1 | 0 | 2,29574257 | 0,688 | 0,022 | 0 | 16 |  |
| ALDH3A1 | 0 | 2,20786518 | 0,592 | 0,003 | 0 | 16 |  |
| KRT5 | 0 | 2,19432927 | 0,606 | 0,011 | 0 | 16 |  |
| ADIRF | 0 | 2,16483102 | 0,761 | 0,003 | 0 | 16 |  |
| FXYD3 | 0 | 2,1446078 | 0,79 | 0,007 | 0 | 16 |  |
| CLDN4 | 0 | 2,10884937 | 0,62 | 0,01 | 0 | 16 |  |
| ID1 | 0 | 2,09077342 | 0,683 | 0,033 | 0 | 16 |  |
| AGR2 | 0 | 2,08226593 | 0,574 | 0,009 | 0 | 16 |  |
| F3 | 0 | 2,02662208 | 0,674 | 0,015 | 0 | 16 |  |
| HES1 | 0 | 1,99232792 | 0,629 | 0,049 | 0 | 16 |  |
| KRT15 | 0 | 1,94538095 | 0,478 | 0,003 | 0 | 16 |  |
| PMCH | 0 | 2,02816231 | 0,23 | 0,003 | 0 | 17 | ILC |
| KLRB1 | 5,906E-178 | 1,67933258 | 0,936 | 0,334 | 1,981E-173 | 17 |  |
| PLIN2 | 1,1655E-72 | 1,64454555 | 0,623 | 0,263 | 3,9088E-68 | 17 |  |
| GATA3 | 6,18E-197 | 1,56497187 | 0,69 | 0,14 | 2,073E-192 | 17 |  |
| IL13 | 6,658E-120 | 1,52930859 | 0,377 | 0,059 | 2,233E-115 | 17 |  |
| XCL1 | 9,462E-45 | 1,52005756 | 0,342 | 0,107 | 3,1734E-40 | 17 |  |
| CSF2 | 7,235E-227 | 1,28716198 | 0,268 | 0,017 | 2,426E-222 | 17 |  |
| FFAR3 | 9,302E-259 | 1,20740021 | 0,256 | 0,013 | 3,12E-254 | 17 |  |
| PHLDA1 | 3,9681E-80 | 1,17999846 | 0,754 | 0,34 | 1,3308E-75 | 17 |  |
| ICOS | 1,449E-120 | 1,14924652 | 0,591 | 0,145 | 4,859E-116 | 17 |  |
| PPARG | 0 | 1,13619774 | 0,425 | 0,016 | 0 | 17 |  |
| TNFRSF18 | 6,126E-181 | 1,12950093 | 0,796 | 0,191 | 2,054E-176 | 17 |  |
| BIRC3 | 7,2804E-48 | 0,93632112 | 0,824 | 0,518 | 2,4417E-43 | 17 |  |
| GADD45G | 7,887E-194 | 0,93023926 | 0,383 | 0,041 | 2,645E-189 | 17 |  |
| NR3C1 | 5,9037E-49 | 0,91900721 | 0,687 | 0,395 | 1,98E-44 | 17 |  |
| IL32 | 2,3464E-51 | 0,91652582 | 0,843 | 0,564 | 7,8694E-47 | 17 |  |
| TCN1 | 0 | 0,90312565 | 0,163 | 0,003 | 0 | 17 |  |
| IL7R | 2,8714E-90 | 0,89891846 | 0,869 | 0,33 | 9,63E-86 | 17 |  |
| IL2RA | 2,159E-197 | 0,87480384 | 0,428 | 0,049 | 7,239E-193 | 17 |  |
| LTB | 5,0617E-18 | 0,87265937 | 0,575 | 0,36 | 1,6976E-13 | 17 |  |
| SLAMF1 | 1,142E-107 | 0,85498385 | 0,45 | 0,094 | 3,831E-103 | 17 |  |
| RORA | 3,4709E-63 | 0,84895065 | 0,744 | 0,359 | 1,1641E-58 | 17 |  |
| IL5 | 1,9547E-73 | 0,83829292 | 0,262 | 0,044 | 6,5556E-69 | 17 |  |
| DUSP4 | 1,3869E-30 | 0,81942441 | 0,591 | 0,334 | 4,6514E-26 | 17 |  |
| PLN | 0 | 0,81617114 | 0,288 | 0,001 | 0 | 17 |  |
| TNFRSF4 | 1,4323E-80 | 0,81103298 | 0,53 | 0,151 | 4,8035E-76 | 17 |  |
| RBPJ | 5,0424E-52 | 0,74951945 | 0,623 | 0,281 | 1,6911E-47 | 17 |  |
| ALAS1 | 1,681E-45 | 0,73555456 | 0,364 | 0,114 | 5,6379E-41 | 17 |  |
| BATF | 1,3241E-58 | 0,73336365 | 0,489 | 0,161 | 4,4409E-54 | 17 |  |
| LMO4 | 9,094E-51 | 0,72495041 | 0,581 | 0,247 | 3,0499E-46 | 17 |  |
| STMN1 | 5,065E-259 | 2,22946101 | 0,948 | 0,216 | 1,699E-254 | 18 | proLy |
| TYMS | 0 | 1,55817481 | 0,747 | 0,01 | 0 | 18 |  |
| TUBA1B | 2,9388E-79 | 1,55577002 | 0,914 | 0,586 | 9,8563E-75 | 18 |  |
| TUBB | 3,5101E-85 | 1,52958535 | 0,796 | 0,355 | 1,1772E-80 | 18 |  |
| HMGB2 | 6,5541E-93 | 1,52474368 | 0,855 | 0,393 | 2,1981E-88 | 18 |  |
| DUT | 3,322E-142 | 1,34464261 | 0,859 | 0,291 | 1,114E-137 | 18 |  |
| HIST1H4C | 1,3008E-20 | 1,29988413 | 0,784 | 0,53 | 4,3628E-16 | 18 |  |
| PCLAF | 0 | 1,25638183 | 0,602 | 0,01 | 0 | 18 |  |
| HMGN2 | 2,4008E-84 | 1,23793713 | 0,914 | 0,602 | 8,0518E-80 | 18 |  |
| GNLY | 1,9689E-59 | 1,20537094 | 0,691 | 0,269 | 6,6032E-55 | 18 |  |
| PCNA | 1,286E-259 | 1,19486696 | 0,721 | 0,1 | 4,312E-255 | 18 |  |
| XCL2 | 2,587E-111 | 1,17930504 | 0,673 | 0,162 | 8,676E-107 | 18 |  |
| MCM7 | 0 | 1,14994274 | 0,725 | 0,059 | 0 | 18 |  |
| H2AFZ | 9,8118E-88 | 1,10697964 | 0,959 | 0,62 | 3,2907E-83 | 18 |  |
| MKI67 | 0 | 1,00519848 | 0,398 | 0,005 | 0 | 18 |  |
| CTSW | 2,8055E-73 | 0,97390969 | 0,892 | 0,464 | 9,4089E-69 | 18 |  |
| CLSPN | 0 | 0,96894944 | 0,591 | 0,013 | 0 | 18 |  |
| DEK | 1,0448E-79 | 0,96009849 | 0,885 | 0,468 | 3,5042E-75 | 18 |  |
| CKS1B | 1,737E-201 | 0,92172493 | 0,602 | 0,083 | 5,826E-197 | 18 |  |
| HMGB1 | 3,3995E-68 | 0,90816611 | 0,967 | 0,855 | 1,1401E-63 | 18 |  |
| NKG7 | 2,0525E-70 | 0,89704122 | 0,952 | 0,511 | 6,8836E-66 | 18 |  |
| RANBP1 | 3,7577E-98 | 0,89236578 | 0,848 | 0,324 | 1,2603E-93 | 18 |  |
| TK1 | 0 | 0,88430451 | 0,39 | 0,004 | 0 | 18 |  |
| ENO1 | 8,5627E-73 | 0,88426272 | 0,944 | 0,654 | 2,8718E-68 | 18 |  |
| DNMT1 | 5,874E-138 | 0,87813096 | 0,773 | 0,193 | 1,97E-133 | 18 |  |
| CENPF | 7,468E-111 | 0,87794308 | 0,297 | 0,036 | 2,505E-106 | 18 |  |
| TOP2A | 0 | 0,86389129 | 0,335 | 0,008 | 0 | 18 |  |
| GAPDH | 8,0772E-86 | 0,85746015 | 0,989 | 0,902 | 2,7089E-81 | 18 |  |
| UBE2C | 0 | 0,85542601 | 0,253 | 0,001 | 0 | 18 |  |
| ACTB | 7,0188E-84 | 0,85326824 | 0,993 | 0,97 | 2,354E-79 | 18 |  |

**Table S5: Top 30 differential gene expression analysis** (scRNA-seq clustermarker) according to highest log2 fold change using logistic regression with Bonferroni correction for each cluster, as compared to the rest of the dataset in all sequenced polyp samples.

**Table S6: please see separate Excel File**

**Differential gene expression analysis within cell types of single cell RNA sequencing analyses** according to highest log2 fold change and adjusted p-value< 0.05 using logistic regression and Bonferroni correction for EpiC, Mc1, MC2, MC3, MyC1, MyC2, pDC, T1,T2,T3,T4,T5,T6 and Treg comparing AERD and CRSwNP. AERD: aspirin-exacerbated respiratory disease; CRSwNP: chronic rhinosinusitis with nasal polyps
